# Heterotypic immunity against vaccinia virus in an HLA-B*07:02 transgenic mousepox infection model

**DOI:** 10.1038/s41598-020-69897-w

**Published:** 2020-08-05

**Authors:** Amrendra Kumar, Naveen Chandra Suryadevara, Kyle J. Wolf, John T. Wilson, Richard J. Di Paolo, James D. Brien, Sebastian Joyce

**Affiliations:** 1 Department of Veterans Affairs, Tennessee Valley Healthcare System, Nashville, TN USA; 2 Department of Pathology, Microbiology and Immunology, Vanderbilt University Medical Centre, Nashville, TN USA; 3 Department of Chemical and Biomolecular Engineering, Vanderbilt University, Nashville, TN USA; 4 Department of Molecular Microbiology and Immunology, Saint Louis University School of Medicine, St. Louis, MO USA

**Keywords:** Adaptive immunity, Antigen processing and presentation, Infection, Vaccines, Immunology

## Abstract

Vaccination with vaccinia virus (VACV) elicits heterotypic immunity to smallpox, monkeypox, and mousepox, the mechanistic basis for which is poorly understood. It is generally assumed that heterotypic immunity arises from the presentation of a wide array of VACV-derived, CD8^+^ T cell epitopes that share homology with other poxviruses. Herein this assumption was tested using a large panel of VACV-derived peptides presented by HLA-B*07:02 (B7.2) molecules in a mousepox/ectromelia virus (ECTV)-infection, B7.2 transgenic mouse model. Most dominant epitopes recognized by ECTV- and VACV-reactive CD8^+^ T cells overlapped significantly without altering immunodominance hierarchy. Further, several epitopes recognized by ECTV-reactive CD8^+^ T cells were not recognized by VACV-reactive CD8^+^ T cells, and vice versa. In one instance, the lack of recognition owed to a N72K variation in the ECTV C4R_70–78_ variant of the dominant VACV B8R_70–78_ epitope. C4R_70–78_ does not bind to B7.2 and, hence, it was neither immunogenic nor antigenic. These findings provide a mechanistic basis for VACV vaccination-induced heterotypic immunity which can protect against Variola and Monkeypox disease. The understanding of how cross-reactive responses develop is essential for the rational design of a subunit-based vaccine that would be safe, and effectively protect against heterologous infection.

## Introduction

Variolation or vaccinia virus (VACV) vaccination has led to the global eradication of smallpox^1,2^. The cessation of vaccination in the late 1970s has rendered a sizable population vulnerable to virulent human-tropic poxvirus infection. The 2003 monkeypox outbreak in the US Midwest alarmingly attests to this vulnerability, and the ability of poxviruses to spread in previously unexpected ways. VACV infection elicits heterotypic immunity to smallpox, monkeypox, and mousepox^2^. This may not be surprising because VACV, variola virus (VARV), the agency of smallpox, monkeypox virus (MPXV), and ectromelia virus (ECTV), which causes mousepox, are related orthopoxviruses that belong to the Chordopoxvirinae subfamily of Poxviridae^3,4^. Nonetheless, these poxviruses are sufficiently removed from each other phylogenetically as well as in terms of their host range and cell tropism, and pathogenetic mechanisms^3,4^. Whilst VACV infects a variety of mammalian species and derived cells, VARV and ECTV infects only humans and rodents, respectively; crossover to other species is not known. MPXV, on the other hand, not only infects monkeys, but will cause zoonotic infections in humans, as does the rodent-tropic ECTV of laboratory mice^3^. These similarities and differences in the species tropisms between poxviruses allows the development of a mechanistic understanding of the basis/bases for heterotypic immunity induced by VACV vaccination. Outcomes of these studies are critical for the rational design of novel subunit vaccines that mimic the immunological profile induced by the current live attenuated vaccine. The potential to develop a subunit vaccine which would mimic the current vaccine, would significantly improve safety and adherence if there was an outbreak of human-tropic poxvirus disease.

It is generally assumed that protective heterotypic immunity to poxviruses arises from the presentation of a wide array of VACV-derived, CD8^+^ T cell epitopes that share sequence homology with other poxviruses^2,3,5–11^. This assumption is supported by a few studies that have shown that VACV-derived, CD8^+^ T cell epitopes are also recognized by ECTV-reactive CD8^+^ T cells generated in C57BL/6 and BALB/c mice. These studies also found that the recognition pattern of the VACV- and ECTV-reactive CD8^+^ T cells were different, altering immunodominance hierarchy in the two strains tested^12–15^. Thus, differences in host range and pathogenesis may lead to alterations in CD8^+^ T cell specificity and function through the differential display and recognition of CD8^+^ T cell epitopes. How VACV and ECTV generate similar and different antigen-specific CD8^+^ T cell responses provide a roadmap to understand and define protection against human-tropic orthopoxviruses, which can be leveraged to develop safe and effective subunit vaccines.

The above findings were extended further in significant ways in the current study: First, this study focuses on the recognition of HLA-B*07:02 (B7.2)-restricted epitopes as a model vaccine design to protect humans against scourges. As well, B7.2 is a prototype member of the B7 supertype. The B7 supertype includes several HLA-I molecules that collectively make up the most frequent supertype of the 12 HLA-I supertypes^16–19^. Second, a large panel of 75 (46 from poxvirus; and 32 host self-protein) B7.2-restricted peptides were screened in this study^5,6,20^. Third, the heterologous ECTV infection of B7.2^tg^ mouse model was used as a surrogate for smallpox in this study because neither VARV nor MPXV are natural mouse pathogens^3,12–15^. Lastly, in contrast to the C57BL/6 mouse, the B7.2^tg^ mouse may no longer show NK cell-mediated resistance to ECTV infection owing to the B6-*H2K*^*b*^*D*^*b*^ null background of the transgenic mouse^21,22^ lending on a greater reliance on the CD8 T cell response for protection. The new data reported herein supports the prevailing view that VACV-elicited heterotypic immunity to poxviruses arises from the recognition of a wide array of VACV-derived, CD8^+^ T cell epitopes that share homology with other orthopoxviruses. Critically, however, several novel ECTV-reactive, CD8^+^ T cell epitopes were identified that were not recognized by VACV-reactive, CD8^+^ T cells, and vice versa. Overall, this understanding of the mechanics of heterotypic immunity were used to develop and test immunogenicity of a recombinant subunit vaccine, illustrating how such findings can be important for rational subunit-based vaccine design.

## Results

### Multiple epitope discovery in a single tube using binary encoded pB7.2 tetramers

**Figure 1:**
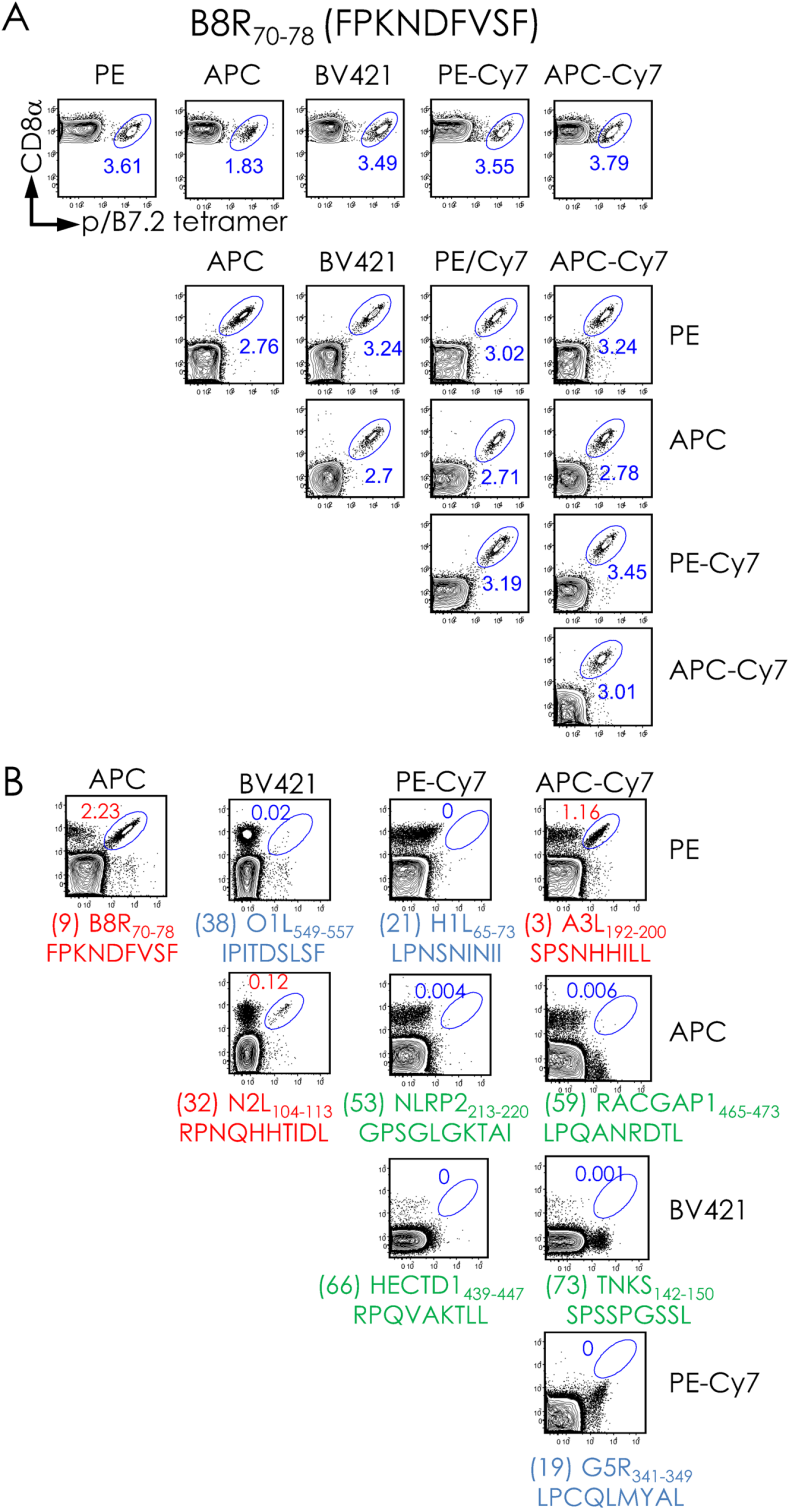
Feasibility of CD8^+^ T cell staining with dual-fluorochrome-encoded pB7.2 tetramers. (**A**) B7^tg^ mice were inoculated i.n. with sublethal dose of VACV, and, after 4 weeks, challenged i.n. with a lethal dose of the virus (see “Materials and methods”). Splenocytes from infected mice were stained with a single fluorochrome-labelled p/B7.2 tetramer (topmost row) or 10 possible two-colour combinations of the B8R_70–78_/B7.2 tetramers in a single staining reaction (lower panels). (**B**) A representative binary encoding strategy querying 10 different specificities in a single reaction using VACV-reactive splenocytes elicited in the experiment described in (**A**) Red, positive VACV pB7.2 tetramer staining; blue, no staining with VACV pB7.2 tetramer; green, no staining with self p/B7.2 tetramers. See Figures S1, S2 for additional binary encoding description and data for tracking 10 distinct T cell specificities in a single pot.

To develop a sample-sparing single pot method for the discovery of multiple CD8^+^ T cell epitopes, we adopted the reported binary-encoded peptide (p)B7.2 tetramer approach^23,24^. To establish this method, B8R_70–78_/B7.2 tetramers were generated with streptavidin tagged with five different fluorochromes (Fig. 1A). The resulting B8R_70–78_/B7.2 tetramers were individually tested against VACV-immune spleen cells that were concurrently stained with anti-CD8α mAb as described previously^23,24^. B8R_70–78_/B7.2 tetramers efficiently stained VACV-reactive CD8^+^ T cells (Fig. 1A, topmost row). All but APC-tagged B8R_70–78_/B7.2 tetramers identified B8R_70–78_-reactive CD8^+^ T cells to the same extent (Fig. 1A, topmost row).

To validate the binary-encoding approach, B8R_70–78_/B7.2 tetramers generated with five different fluorochrome-tagged streptavidin and two fluorochrome-tagged B8R_70–78_/B7.2 tetramers were added to each tube. After staining with anti-CD8α mAb, CD8^+^ T cells bound with B8R_70–78_/B7.2 tetramers that were tagged with the two different fluorophores (binary encoding) were detected by flow cytometry (see Figure S1). As above, all tetramers but for those that included APC-tagged B8R_70–78_/B7.2 tetramer efficiently identified B8R_70–78_-reactive CD8^+^ T cells to the same level (Fig. 1A, rows 2–5). This result established the binary encoding method for this project using a monospecific, B8R_70–78_/B7.2 tetramer.

To establish whether the binary encoding approach will detect multiple specificities in a single tube, the indicated pB7.2 monomers (Figs. 1B, S2) were generated. For this, each of the 75 peptides (see Table S1) were individually loaded onto a conditional pB7.2 monomer that was generated as described previously^7,20,23,24^. Peptides for this assay were chosen based on their ability to replace the UV-labile peptide bound to the conditional pB7.2 monomer (see “Materials and methods”). A ≥ 40% exchange was used as the cut-off because we had previously shown that level of exchange was sufficient to detect a tetramer reactive CD8^+^ T cells from an immune spleen^20^. Further, 46 of the 75 peptides were VACV-derived, and the rest (29 of the 75 peptides) were self-peptides that were bound to B7.2 molecules isolated from VACV-infected HeLa cell line (Table S1)^20,25^. Three of the 46 VACV-derived peptides differed by one amino acid residue from the corresponding VACV-derived epitope but matched the VARV proteome (see red residues indicated in Table S1). These peptides were included to determine whether ECTV infection would elicit CD8^+^ T cells against VARV variants because VACV infection did not^20^. Self peptides were included, one, as a negative control and, two, to determine cross-reactivity toward self^26^.

Each monomer was then tetramerised with two different fluorochrome-tagged streptavidin as indicated (Fig. 1B; see Figures S1, S2). Then binary-encoded pB7.2 tetramers of ten distinct specificities were added to a single tube containing VACV-immune spleen cells. After staining with anti-CD8α mAb, the specificity of CD8^+^ T cells contained in the VACV-reactive spleen cells were ascertained by flow cytometry. This method detected three previously reported VACV-derived CD8^+^ T cell epitopes within VACV-immune spleen cells (marked in red in Figs. 1B, S2)^20^. The frequency of the CD8^+^ T cells were similar to those previously reported (Fig. 1B)^20^. As expected^20^, the remaining seven of the 10 pB7.2 tetramers, which included self-pB7.2 complexes, contained within the panel shown in this figure do not detect VACV-reactive CD8^+^ T cells (marked in blue and green in Figs. 1B, S2). Together, the above results validate the quality and specificity of binary-encoded pB7.2 tetramers.

One cautionary note, however, is that the pB7.2-APC tetramer underestimates the frequency of reactive CD8^+^ T cells by about 15–35% (Fig. 1A). Notwithstanding that, when combined with one of the other four fluorochromes, APC-conjugated tetramers performed just as well as the other fluorochromes and, hence, did not affect the frequency of tetramer positive cells (Fig. 1).

### ECTV-reactive CD8^+^ T cells recognise unique and common VACV-derived epitopes

We previously reported the characterisation of 65 naturally processed, VACV strain Western Reserve (WR)-derived peptides that were presented by B7.2 molecules during an infection^20^. Of the 46 (43 VACV-derived plus 3 VARV variant) peptides, 19 were recognised by CD8^+^ T cells in human peripheral blood mononuclear cells (PBMCs) obtained from six volunteers vaccinated with the smallpox vaccine, DryVax^20^. Further, 14 of the 46 VACV-derived and variant peptides were recognised by B7.2^tg^ (B6-*K*^*0*^*D*^*0*^;B*07:02) mice (see Table 1)^20^. Therefore, it is generally assumed that the success of the small pox vaccine at a population scale owes to the broad heterotypic immunity elicited by the vaccine^2,3,5–7^. To test this assumption, we compared the response of ECTV-specific CD8^+^ T cells and those against VACV to VACV-derived peptides.

**Table 1:** Previously reported VACV-derived CD8^+^ T cell epitopes presented by B7.2: their homology to ECTV proteome and recognition by CD8^+^ T cells.

VACV ORFs^a^	VACV epitopes^b^	ECTV^c^	Recognition by		
			VACV-reactive T cells^b,d^	ECTV-reactive T cells^e^	hu PBMCs^f^
D1R_808–817_	RPSTRNFFEL	•	+	+	+
A34R_82–90_	LPRPDTRHL	•	+	+	**–**
D1R_686–694_	HPRHYATVM	•	+	+	+
J6R_303–311_	MPAYIRNTL	•	+	+	+
F4L_6–14_	APNPNRFVI	•	+	+	**–**
E2L_216–224_	RPRDAIRFL	•	+	+	+
A3L_192–200_	SPSNHHILL	•	+	+	+
D5R_375–383_	LPKEYSSEL	•	+	+	**–**
L4R_37–45_	FPRSMLSIF	•	+	+	+
E9L_526–534_	FPYEGGKVF	•	+	+	+
I6L_282–291_	IPKKIVSLL	•	**–**	+	**–**
B15R_91-101_	IPDEQKTIIGL	•	**–**	+	**–**
D9R_26-35_	IPRSKDTHVF	•	**–**	+	**–**
A24R_1002-1010_	KPYASKVFF	•	**–**	+	**–**
B8R_70–78_^ g^	FPKNDFVSF	K72N	+	**–**	+
N2L_104–113_	RPNQHHTIDL	Q107K	+	**–**	+
O1L_335–344_	RPMSLRSTII	NSH	+	**–**	+
A4L_126–135_	APASSLLPAL	NSH	**–**	**–**	+
B22R_72-80_	TVADVRHCL	NSH	**–**	**–**	+
E9L_175–183_	FPSVFINPI	•	**–**	**–**	+
A11R_22–30_	YPSNKNYEI	•	**–**	nt	+
A20R_162–170_	IPKYLEIEI	•	**–**	nt	+
C19L/B25R_78–86_	NPSVLKILL	N78K	**–**	nt	+
E5R_131-1–40_	NPSKMVYALL	•	**–**	nt	+
G2R_140-149_	VPITGSKLIL	NSH	**–**	nt	+
I6L_237–245_	FPTNTLTSI	•	+	nt	**–**
I6L_282–291_	LPSNVEIKAI	•	**–**	nt	+

^a^Open reading frames and location of epitopes defined based on Copenhagen reference strain (VACCC, ID 10,249).

^b^VACV-derived epitopes recognised by CD8^+^ T cells reported by us and others in mice and humans^20^.

^c^Amino acid changes in the orthologous ECTV peptide; •, conserved sequences with 100% homology; NSH, no significant homology; based on Netblast (blastcl3: www.ncbi.nlm.nih.gov) using ECTV txid12643.

^d^B7.2^tg^ mouse CD8^+^ T cells that react to VACV^20^.

^e^B7.2^tg^ mouse CD8^+^ T cells that react to ECTV (this study): + , positive response (see Fig. 2); –, no response; nt, not tested.

^f^hu, VACV-reactive CD8^+^ T cells in human PBMC^20^.

^g^B8R orthologue in ECTV is C4R based on Netblast using ECTV txid12643.

We first compared the amino acid sequences of VACV-derived CD8^+^ T cell epitopes that are presented by B7.2 molecules during a natural infection with the homologous region in the ECTV proteome by in silico analysis. Homologies between VACV and ECTV proteome in the region of VACV-reactive CD8^+^ T cell epitopes were identified using Netblast (blastcl3: www.ncbi.nlm.nih.gov) using ECTV, txid12643, as the taxonomic id. We found that three of the VACV epitopes to vary in ECTV by a single amino acid change (Table 1). Further, four of the VACV epitopes had no significant homology (NSH) to the orthologous region in ECTV (Table 1). Hence, we predict that these variations may impact VACV epitopes recognised by the heterologous ECTV-specific CD8^+^ T cells.

To test the above prediction, we elicited ECTV-specific CD8^+^ T cell response by intranasal (i.n.) inoculation (priming) of B7.2^tg^ mice with 10 pfu ECTV. Mice were boosted i.n. 30 days after prime with 200 pfu ECTV. Simultaneously, VACV-specific CD8^+^ T cells were generated as reported previously^20^. Mock (PBS)-treated mice served as the negative control. Eight days post boost, spleen mononuclear cells were prepared and the specificity of the pox virus-specific CD8^+^ T cells was ascertained using binary-encoded fluorescent pB7.2 tetramers prepared as described above (Fig. 1B). All 75 VACV peptides, those reported by us and others, were individually loaded onto a conditional pB7.2 monomer as described previously^5,6,20,23,24^. Thus, the use of five different fluorophores for binary encoding allow the discovery of 10 distinct specificities in a single reaction (Fig. 1A).

**Figure 2:**
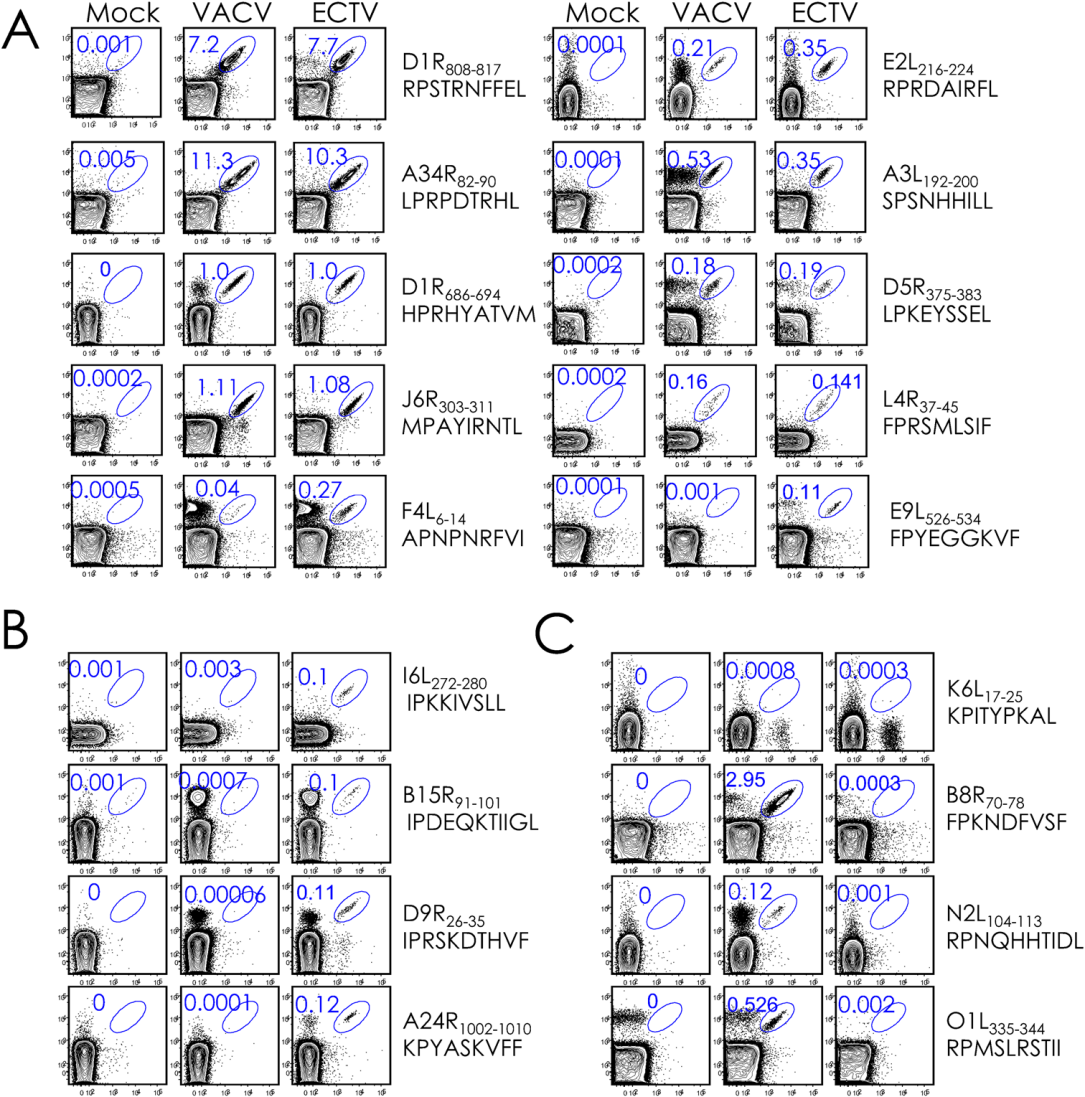
Tracking VACV- and ECTV-reactive CD8^+^ T cell responses in B7.2^tg^ mice. B7^tg^ mice were inoculated i.n. with either PBS or either a sublethal dose of VACV or ECTV. After 4 weeks, primed mice were challenged i.n. with a lethal dose of the same virus (see “Materials and methods”). Spleens were harvested from mock (n = 2), VACV (*n* = *2*) inoculated mice after 8–10 days post inoculation. CD8^+^ T cells were identified as CD8^+^ splenocytes after gating out dead cells and B220^+^ cells. Peptide-specific CD8^+^ T cells were identified with dual fluorochrome-labelled pB7.2 tetramers and binary encoding strategy (“Materials and methods”, and Fig. 1, and Figures S1, S2). Representative contour plots depicting common epitopes recognised by VACV and ECTV-reactive CD8^+^ T cells (**A**), novel epitopes F4L_6-14_ and E9L_526-534_ recognised by ECTV- but not VACV-reactive CD8^+^ T cells (**B**), and epitopes recognised by VACV- but not ECTV-reactive CD8^+^ T cells (**C**).

**Figure 3:**
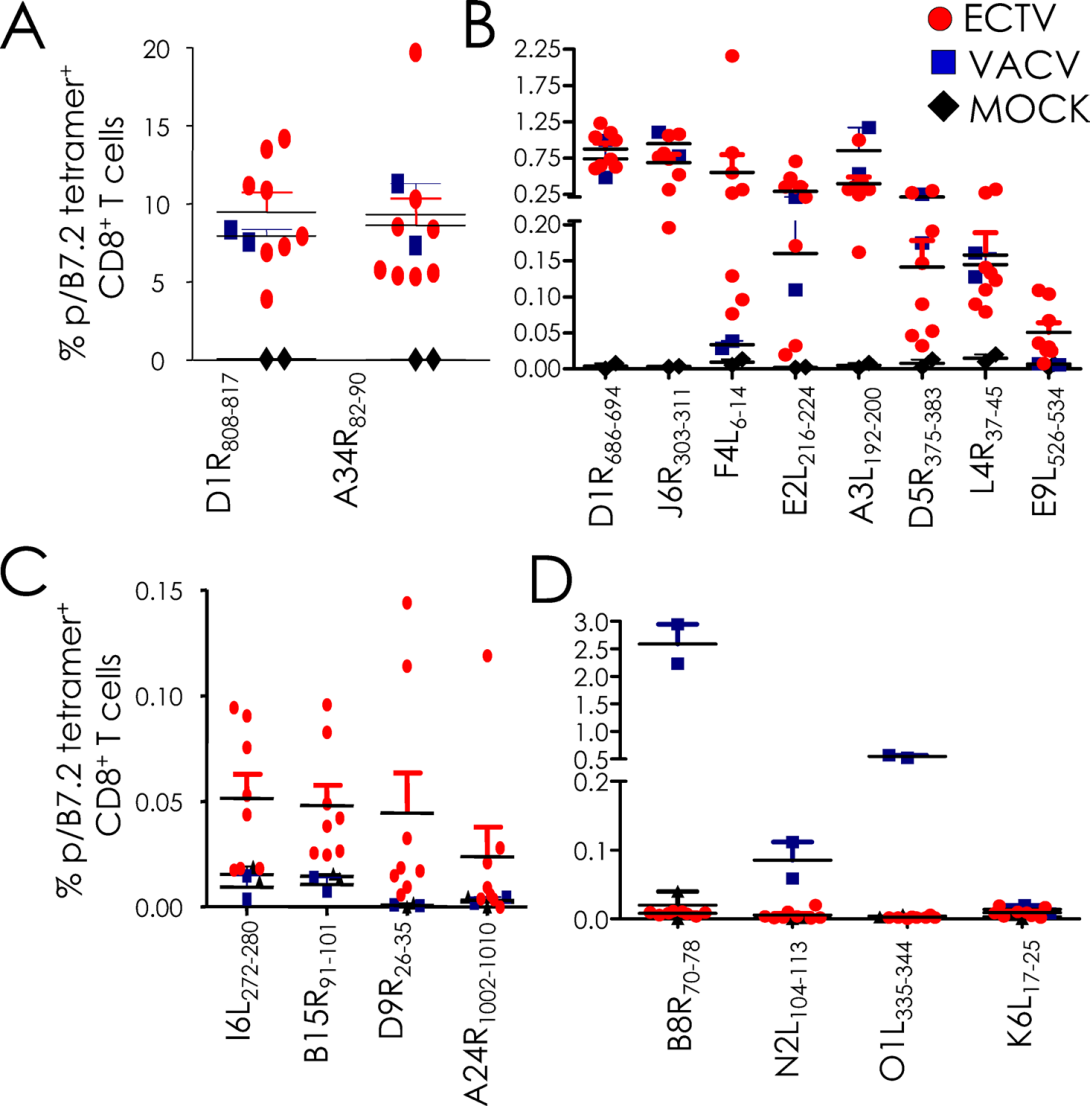
Quantification of VACV- and ECTV-reactive CD8^+^ T cells responses in B7.2^tg^ mice. Frequencies (% of CD8^+^) ECTV- and VACV-specific CD8^+^ T cells defined by epitope specific pB7.2 tetramer staining of splenocytes from infected animals based on the experiment in Fig. 2. Background staining was determined by irrelevant HMPV-derived CD8^+^ T cell-reactive epitope B7.2 tetramer binding or by staining of mock infected samples. Data are mean ± standard error of the mean (sem); ECTV (*n* = *8*), VACV (*n* = *2*) or mock (*n* = *2*).

pB7.2 tetramer-based analysis revealed that the ECTV infection elicited CD8^+^ T cells to 10 of the 14 VACV-reactive CD8^+^ T cell epitopes (Fig. 2A). This response mirrored the VACV-elicited CD8^+^ T cell response both in specificity and in frequency (Figs. 2A, 3A,B). Surprisingly, ECTV-specific CD8^+^ T cells recognised four new epitopes that were not recognised by VACV-reactive CD8^+^ T cells (Figs. 2B, 3C) despite the fact that the ECTV- and VACV-derived peptides were cent-per-cent identical to each other in all four cases (Table S2). The response to the four new epitopes was of low frequency (Figs. 2B, 3C). As well, ECTV did not elicit CD8^+^ T cell responses to three subdominant epitopes recognised by VACV-reactive CD8^+^ T cells (Figs. 2C, 3D). Background levels of pB7.2 tetramer reactivity toward splenocytes from mock-treated mice, and the lack of reactivity to the K6L_17–25_ peptide and any of the 32 self peptides by ECTV- and VACV-specific CD8^+^ T cells attested to the specificity of the responses described above [Figs. 1 (marked in green), 2, 3].

### Variation in pox virus proteomes impacts epitope specificity of virus-specific CD8^+^ T cells

**Figure 4:**
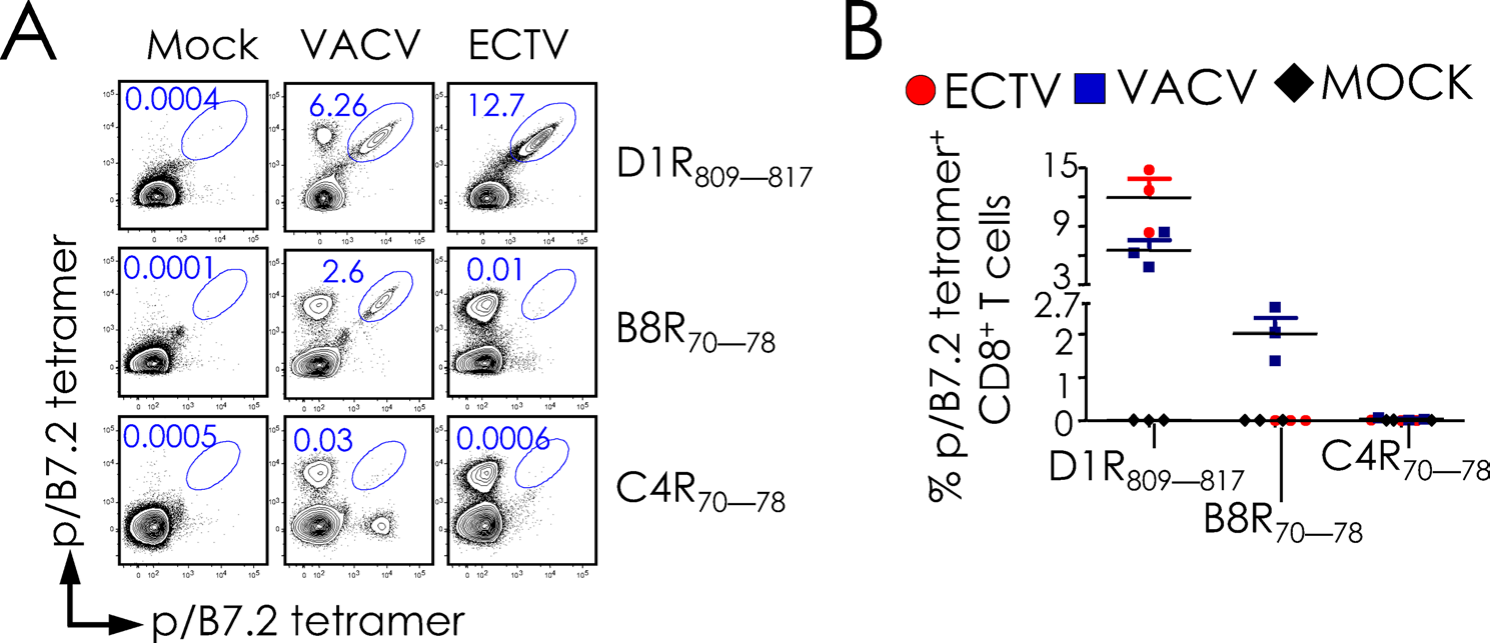
ECTV-reactive CD8^+^ T cells does not recognise the C4R_70–78_ peptide, the ortholog of B8R_70–78_. (**A**) B7.2^tg^ mice were inoculated i.n. with VACV as in Fig. 1. Spleens were harvested from mock (*n* = *3*), ECTV (*n* = *3*) or VACV (*n* = *3*) inoculated mice 8–10 days after secondary infection. CD8^+^ T cell responses were monitored using pB7.2 tetramers using dual color labelling and multi-dimensional encoding strategy as in Fig. 1. Representative contour plots depicting CD8^+^ T cell response to B8R_70–78_ and C4R_70–78_ in ECTV- and VACV- inoculated splenocytes. CD8^+^ T cell response to common epitope D1R_808-817_, served as positive control. (**B**) Frequencies (% of CD8^+^) of B8R_70–78_, C4R_70–78_ or D1R_808-817_ (positive control) reactive CD8^+^ T cells in VACV-specific CD8^+^ T cells defined by pB7.2 tetramer staining in infected splenocytes. Data are mean ± sem; *n* as in (**A**).

As B8R_70–78_, N2L_104–113_, and O1L_335–344_ peptides in ECTV vary (Table 1), we considered the possibility that the respective VACV-derived peptides are not recognised by the ECTV-reactive CD8^+^ T cells. To test this possibility, we focused on the C4R_70–78_ peptide from ECTV because the orthologous B8R_70–78_ peptide of VACV that differs by a single amino acid residue N72K, is one of the dominant VACV-reactive CD8^+^ T cell epitopes and confers protection from lethal respiratory VACV infection^20,27^. So also, the ECTV ortholog of the VACV N2L_104–113_, peptide differs only by a single residue: (Table 1); it was not scrutinised as it is a subdominant epitope^20^. In contrast, the ECTV ortholog of VACV O1L_335–344_ peptide has no significant homology (Table 1) [based on Netblast (blastcl3: www.ncbi.nlm.nih.gov) using ECTV [txid12643], and was not studied further. Hence, the reaction of D1R_808–817_/B7.2 as positive control, and C4R_70–78_/B7.2, and B8R_70–78_/B7.2 tetramers toward ECTV- and VACV-specific CD8^+^ T cells were determined. As expected D1R_808–817_/B7.2 tetramer reacted to both ECTV- and VACV-specific CD8^+^ T cells, whilst the B8R_70–78_/B7.2 tetramer reacted only to VACV-specific CD8^+^ T cells but not to ECTV-reactive CD8^+^ T cells (Fig. 4). Surprisingly, however, C4R_70–78_/B7.2 tetramer reacted to neither ECTV- nor VACV-specific CD8^+^ T cells (Fig. 4).

**Figure 5:**
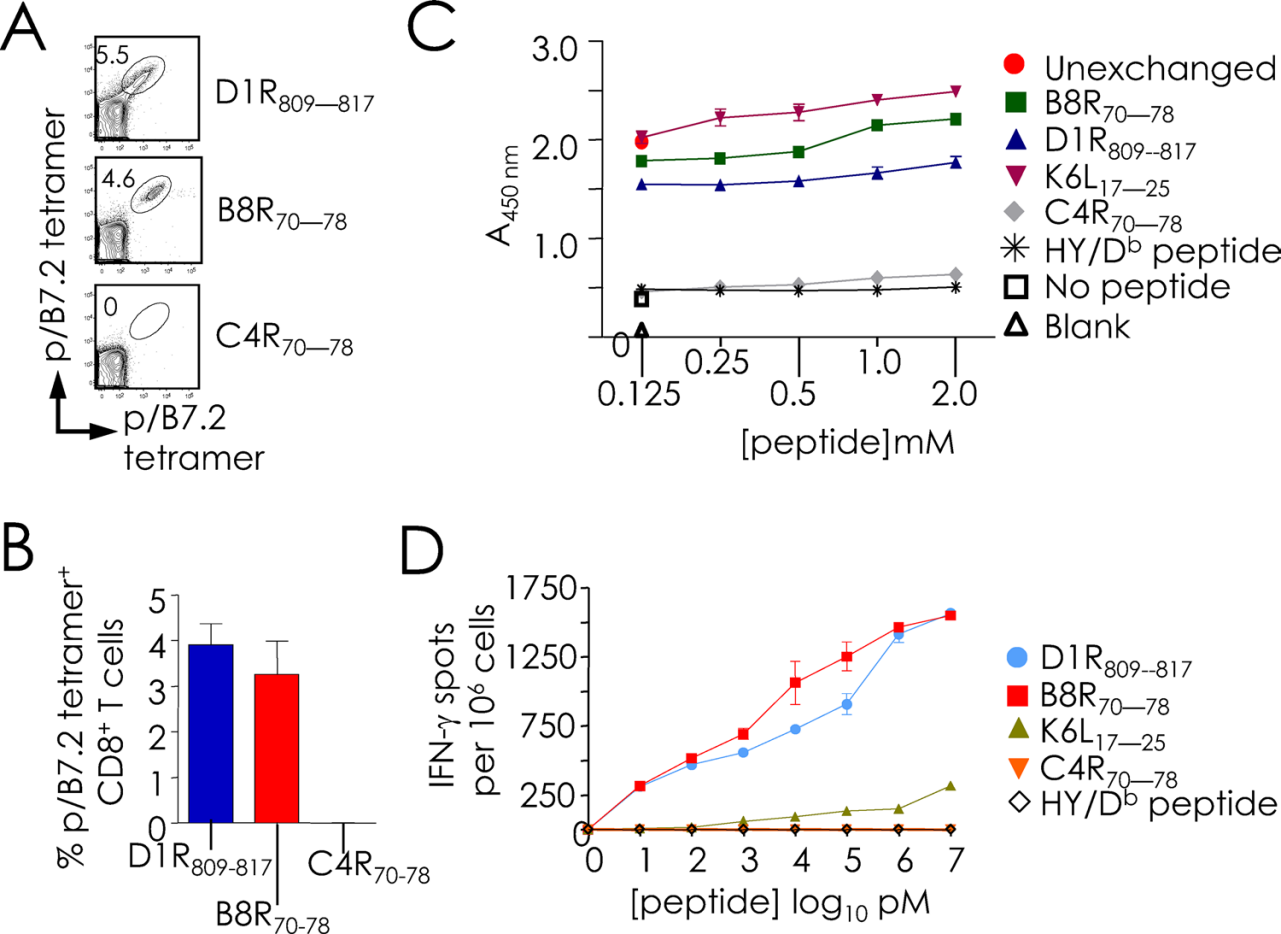
B8R_70–78_ peptide of VACV is mutated in ECTV and does not elicit CD8^+^ T cell responses. B7.2^tg^ mice (*n* = *6*) were primed with a mixture of peptides using TriVax vaccination protocol and boosted 14 days later with maturated BMDCs that were pulsed with the indicated peptides as described in “Materials and methods” to elicit peptide-specific CD8^+^ T cell responses. Ten days after boost, splenocytes were harvested, and peptide-reactive CD8^+^ T cell responses were monitored. (**A**) Representative contour plots (*n* = *6*) depicting D1R_808-817_, B8R_70–78_ and C4R_70–78_ specific CD8^+^ T cell responses that were detected with the indicated pB7.2 tetramers, each of which was labelled with two different fluorochromes. Numbers in oval gates correspond to the frequency of antigen specific CD8^+^ T cells. (**B**) Frequencies (% of CD8^+^) of B8R_70–78_, C4R_70–78_ or D1R_808-817_ specific CD8^+^ T cells were enumerated with pB7.2 tetramer staining in infected spleocytes. Data are mean ± sem; *n* as in (**A**). (**C**) Peptide binding to conditional pB7.2 monomer was measured by the ability of the exchange peptide to stabilize HLA-I. Soluble AARG-**J**-TLAM/B7.2 monomer was incubated with the peptides indicated at the concentrations shown and subjected to a 60-min UV radiation. No UV (un-exchanged) AARG-**J**-TLAM/B7.2 monomer was used as a positive control and monomer without any peptide served as negative/background control. Peptide exchange-stabilized pB7.2 was monitored by ELISA by detecting human β2-m bound to MHC-I tethered to the ELISA plates using the pan-HLA-I mAb W6/32. Data are mean ± sem of exchange performed in triplicate for each peptide concentration, and representative of three independent experiments. (**D**) Dose dependent IFN-γ response elicited by TriVax primed and peptide-pulsed BMDC-boosted mouse splenocytes that was measured by epitope titration in an ELISpot assay. Data represent the mean of triplicate wells; representative of two independent experiments. *n* = *3* mice in each experiment.

This pB7.2 tetramer reaction pattern could have arisen from, one, a lack of CD8^+^ T cell repertoire against C4R_70–78_ in B7.2^tg^ mice, or, two, to poor or no binding of C4R_70–78_ to B7.2. To test the first possibility, B7.2^tg^ mice were primed with a mixture of D1R_808–817_, B8R_70–78_, and C4R_70–78_ peptides mixed with anti-CD40 mAb + poly-I:C as the adjuvant, and then boosted by intraperitoneal (i.p.) route with bone marrow derived dendritic cells (BMDCs)-pulsed in vitro with the three peptides two weeks post priming. Two weeks post boost, immune spleen cells were tested for peptide specific responses by tetramer staining and interferon (IFN)-γ ELISpot assay. Tetramer staining detected D1R_808–817_- and B8R_70–78_-reactive CD8^+^ T cells as expected (Fig. 5A,B). But surprisingly, B7.2^tg^ mice did not respond to the C4R_70–78_ peptide (Fig. 5A,B). Hence, we predicted that the ECTV C4R_70–78_ peptide does not bind to B7.2 owing to the K72N variation in the ECTV peptide when compared to the orthologous VACV B8R_70–78_ peptide. Or, alternatively, the B7.2^tg^ mouse lacks a C4R_70–78_-reactive CD8^+^ T cell repertoire.

To directly test the first possibility, varying concentrations of D1R_808–817_, B8R_70–78_, C4R_70–78_, and K6L_17–25_ peptides, and an irrelevant H-2D^b^-restricted epitope were exchanged with the conditional peptide-bound B7.2 monomer as described above. The D1R_808–817_ and K6L_17–25_ peptides exchanged with the conditional p/B7.2 monomer just as well as the B8R_70–78_ peptide (Fig. 5C). Nonetheless, C4R_70–78_ peptide did not exchange with the conditional pB7.2 monomer (Fig. 5C). Hence, it is unlikely that the B7.2^tg^ mouse does not have a C4R_70–78_-reactive CD8^+^ T cell repertoire. This notion was confirmed in a functional assay by probing with peptide-immune spleen cells that were derived from mice primed and boosted with a mixture of D1R_808–817_, B8R_70–78_, and C4R_70–78_ peptides. Thus, only D1R_808–817_ and B8R_70–78_ peptides but not the C4R_70–78_ peptide stimulated an Interferon (IFN)-γ response in vitro (Fig. 5D). Hence, because of the inability of the C4R_70–78_ peptide to bind to B7.2 molecule, it is neither immunogenic nor antigenic. Further, the above data taken together suggest that sequence variations in poxvirus proteomes can impact CD8^+^ T cell responses.

### Recombinant subunit vaccine, engineered to express the D1R_808–817_, B8R_70–78_, and C4R_70–78_ epitopes, plus αGalCer elicit robust CD8^+^ T cell responses to two of the three peptides

**Figure 6:**
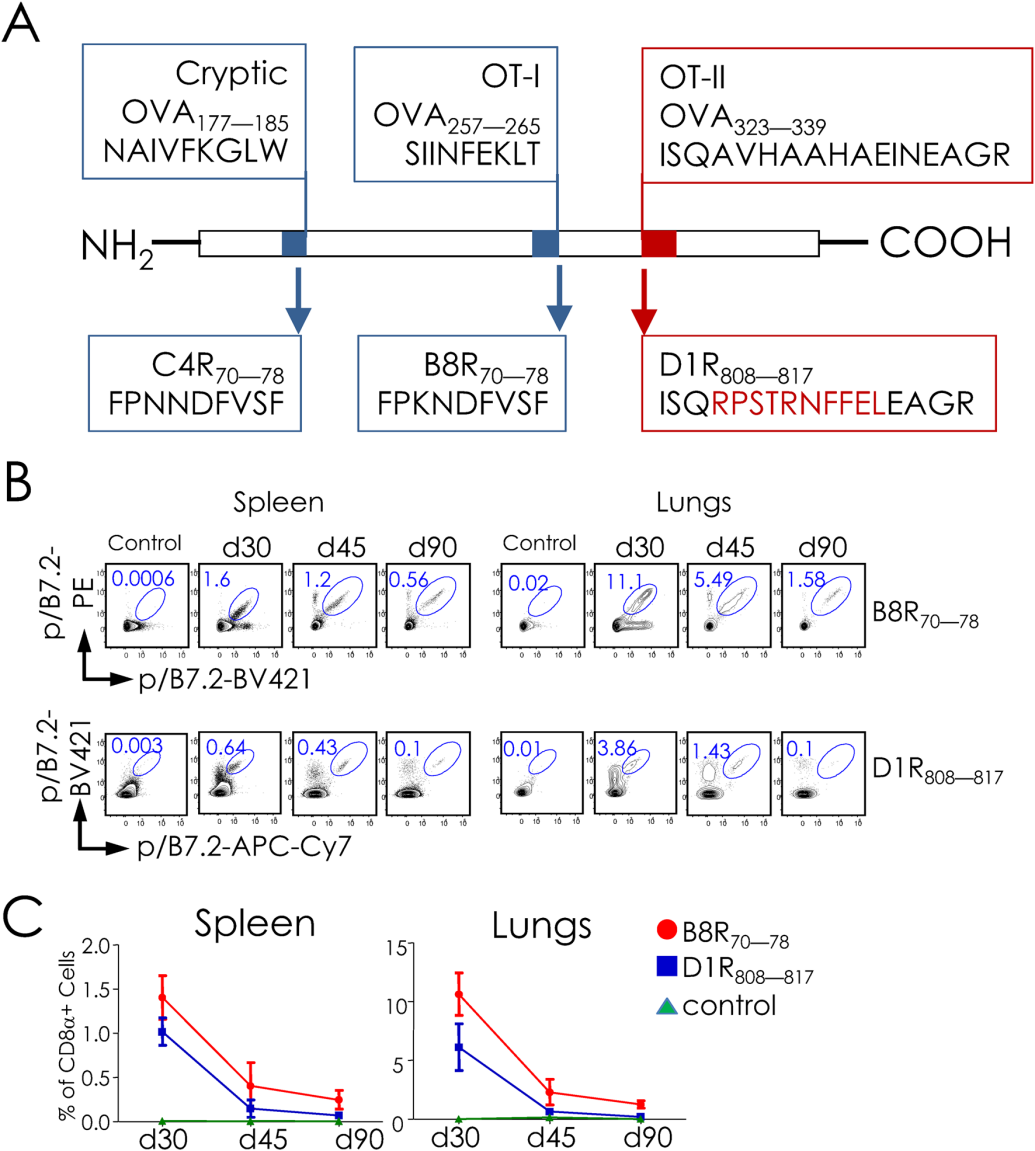
Intranasal vaccination with rOVA-3 plus αGalCer elicits CD8^+^ T cell responses. (**A**) Schematics of OVA (rOVA-3) construct in which the original cryptic, OT-I and OT-II epitopes were replaced with C4R_70–78_, B8R_70–78_ and D1R_808–817_ epitopes, respectively. rOVA-3 containing a C-terminal hexa-histidine tag was constructed by gene synthesis and cloned into pET24a for expression in *E. coli*. (**B,C**) B7.2^tg^ mice were primed i.n. with rOVA-3, which contains the D1R_808–817_ and B8R_70–78_ epitopes, mixed with αGalCer or αGalCer alone as the control without rOVA-3. After 14 days, primed mice were similarly boosted. Spleens and lungs were harvested on day 30, 45, and 90 after boost. D1R_808–817_ and B8R_70–78_ specific CD8^+^ T cell responses were monitored using epitope-specific pB7.2 tetramers as in Fig. 1. (**B**) Representative contour plots show D1R_808–817_- and B8R_70–78_-specific CD8^+^ T cells derived from 2–3 independent experiments with two mice in each group/time point. Numbers represent frequency of indicated peptide-specific D1R_808–817_ and B8R_70–78_ specific CD8^+^ T cells at the indicated time points. (**C**) Frequency (% of CD8^+^) of D1R_808–817_- and B8R_70–78_-specific CD8^+^ T cells defined by pB7.2 tetramer staining in subunit vaccinated splenocytes and lung leukocytes. Data are cumulative mean ± sem; day 30 (*n* = *6*), day 45 (*n* = *4*), day 90 (*n* = *4*) or control (*n* = *4*).

Next we established a model that yields CD8^+^ T cell responses to multiple epitopes from a single protein subunit. We previously engineered VACV-derived L4R protein to express the CD8^+^ T cell-mediated protective epitope B8R_70–78_. As the L4R scaffold accommodates only one epitope, we engineered ovalbumin (OVA) protein, which has two significant advantages: One, OVA is much more soluble when compared to L4R after expression in *E. coli* and refolding in vitro. Two, there are two H-2^b^ class I- (OT-I and a cryptic CD8^+^ T cell epitope) and one H-2A^b^ (OT-II)-restricted epitopes in OVA. The focus was on D1R_808–817_- and B8R_70–78_-specific CD8^+^ T cells because they were generated in high frequency after peptide and protein vaccination, and are protective against i.n. lethal VACV infection^20,27^. C4R_70–78_ was used as the specificity control. Hence, we replaced all three T cell epitopes in OVA with D1R_808–817_ [in place of H-2K^b^-restricted OT-I (Ova_257–264_)], B8R_70–78_ [replacing nine amino acid residues of H-A^b^-restricted OT-II (Ova_323–339_)], and C4R_70–78_ [substituting the cryptic H-2D^b^-restricted epitope (Ova_177–185_)] resulting in recombinant rOVA-3 (Fig. 6A; see “Materials and methods”). Similarly, a control rOVA-HY, which substitutes OT-II epitope with the H-2A^b^-restricted male antigen (HY)-derived CD4^+^ T epitope^28,29^ was constructed (Figure S3A). Expression of rOVA-3 and the control rOVA-HY resulted in proteins of Mr ~ 43 kDa (Figure S3B).

Prime-boost vaccination 14 days apart with rOVA-3 plus the natural killer T (NKT) cell agonist αGalCer as the adjuvant^20,27,30–32^ by i.n. route resulted in B8R_70–78_ and D1R_808–817_ epitope-specific CD8^+^ T cell responses in the lungs and the spleen (Fig. 6B). As previously reported by us^27^, the response to B8R_70–78_ by subunit vaccination was robust at day 30 post boost that waned by day 45 post boost and was low by day 90 post boost. Similar responses to the D1R_808–817_ epitope was also observed that waned much more rapidly (Fig. 6B,C). This result prompted us to identify and phenotype CD8^+^ tissue-resident memory (TRM) T cells that were elicited in the lungs by i.n. prime boost vaccination with rOVA-3.

### Phenotyping of CD8^+^ T cells isolated from the lungs suggests the elicitation of TRM cells

**Figure 7:**
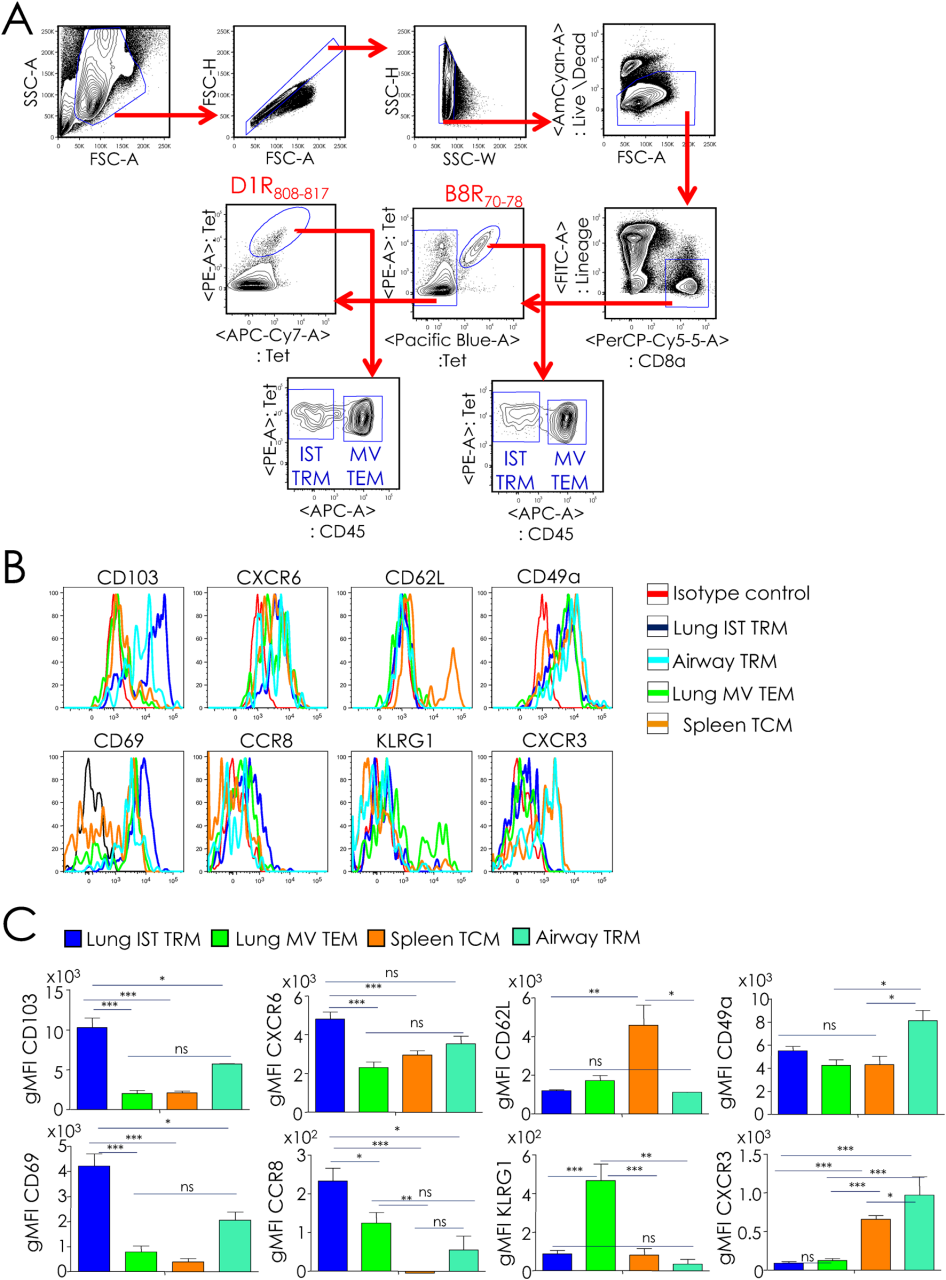
Intranasal delivery of rOVA-3 subunit vaccine plus αGalCer elicits CD8^+^ TRM responses. B7.2^tg^ mice (*n* = *5*) were primed and boosted i.n. as in Fig. 6. On day 30 after boost, mice were injected i.v. with anti-CD45.2-APC mAb. After 3–5 min., airway cells were collected by perfusing the lungs with PBS. Airway, lungs and spleen single cell suspensions were stained for surface markers and antigen specificity, and gated to identify B8R_70–78_-specific, CD8^+^ TRM cells in the airways and lung interstitium (IST TRM), lung CD8^+^ MV TEM cells, and splenic TCM cells. Expression of surface activation markers was assessed on the indicated populations. Representative gating strategy (**A**), overlay histograms (**B**), and geometric mean fluorescent intensity (gMFI) of surface indicated markers on specified populations (**C**) are shown. Data are cumulative mean ± sem. Airway TRM (*n* = 2); and IST TRM; MV TEM, TCM: *n* = 5. ns, not significant (*P* > 0.05), *P ≤ 0.05, **P ≤ 0.001, ***P ≤ 0.0001.

Phenotypic evaluation of B8R_70–78_ and D1R_808–817_ epitope-specific CD8^+^ lung interstitial (anti-CD45^NEG^: IST and airway TRM), marginated vascular (anti-CD45^POS^: MV TEM) and splenic (TCM) memory T cells was performed after intravital staining of leukocytes with anti-CD45.2 mAb for 3–5 min. prior to tissue harvest as described previously^27^. Anti-CD45^NEG^, IST CD8^+^ T cells isolated from the lungs expressed high levels of CD69 and CD103, when compared to anti-CD45^+^, MV CD8^+^ T cells, which stained poorly with the mAb against CD103, but stained at intermediate levels with anti-CD69 mAb (Fig. 7). On the other hand, anti-CD45^NEG^, CD8^+^ T cells from the airways expressed intermediate levels of CD69 and CD103 when compared to IST CD8^+^ T cells, whilst splenic T cells were negative for both markers (Fig. 7). Thus, rOVA-3 prime boost vaccination elicited CD8^+^ IST TRMs in the lungs.

Consistent with the installation of CD8^+^ T cells in the lung tissue, IST CD8^+^ TRM T cells expressed lower levels of KLRG1 and CD62L when compared to other antigen-specific CD8^+^ T memory subsets^27^. Further, lung IST TRM T cells but not the other memory cells elicited by rOVA-3 via i.n. prime boost vaccination expressed high levels of the chemokine receptors CXCR6 and CCR8 (Fig. 7). In contrast, airway CD8^+^ TRM T cells but not the other memory cells expressed CXCR3 and CD49a (Fig. 7). On the bases of these phenotyping data, we conclude that rOVA-3 is immunogenic and generates lasting lung-resident (airway and IST TRMs) and peripheral (MV TEMs and TCMs) memory CD8^+^ T cells.

## Discussion

In summary, we found that ECTV- and VACV-reactive CD8^+^ T cells recognise an overlapping subset of the dominant VACV-derived epitopes, whilst others were recognised by one but not the other effector T cells. These findings have implications for vaccine design as demonstrated by the non-immunogenic and non-antigenic nature of the C4R_70–78_ peptide derived from ECTV. We further demonstrated that epitopes recombinantly expressed via an OVA scaffold (rOVA-3), as a model subunit vaccine, resulted in the generation of lung-resident CD8^+^ T cells that possessed all the phenotypic features of lung IST TRM cells.

Efforts from several groups have led to the discovery and characterization of numerous VACV-derived CD8^+^ T cell epitopes. Most studies have used homology of these epitopes to other poxviruses, especially VARV, ECTV, and MPXV proteomes as the basis for heterotypic immunity^5–13,15,20,25^. Only rarely was this conjecture put to a comprehensive test^12,13,33^. Hence, herein we tested the assumption that heterotypic immunity arises from the presentation of a wide array of VACV-derived, CD8^+^ T cell epitopes that share homology with other poxviruses. The ECTV infection of mice was used as the model because mousepox pathogenesis is thought to be similar to that of smallpox disease^22^. In a large-scale analysis of poxvirus-derived epitopes reported herein, we found significant overlap in the dominant epitopes recognised by ECTV- and VACV-reactive CD8^+^ T cells. The recognition pattern by the two effector T cell populations were similar without significantly altering the immunodominance hierarchy. This result contrasts that reported for H-2^b^-restricted poxvirus-specific CD8^+^ T cell responses^12,13^. The overlapping epitope recognition pattern reported herein supports the prevailing view that vaccination with VACV elicits heterotypic immunity to poxviruses, and that this type of immunity arises from the recognition of a wide array of VACV-derived, CD8^+^ T cell epitopes that share homology with other orthopoxviruses.

We found that ECTV-reactive CD8^+^ T cells recognised a few novel VACV-derived epitopes but, surprisingly, not by VACV-reactive CD8^+^ T cells even though the four epitopes were identical in the VACV and ECTV proteomes, and presented by B7.2 molecules expressed by VACV-infected HeLa cells^20^. Conversely, a few epitopes recognised by VACV-reactive CD8^+^ T cells were not recognised by ECTV-reactive CD8^+^ T cells. The exact reason for the differential recognition of several epitopes currently remains unclear. One possibility, however, is that variations in the flanking regions of the corresponding orfs (see Table S2) may differentially impact efficient presentation of epitopes in ECTV-infected *versus* VACV-infected cells in vivo. These differences could be further influenced by pathogenesis incited by the two viruses^22^ and, thereby, modify antigen processing, presentation, and recognition. Another possibility is that the in vivo expression pattern of the parent proteins from which such epitopes were derived are different between the two viruses. Such expression patterns may arise from polymorphisms in the gene regulatory regions that drive the expression of the *orf*s. These possibilities need to be formally ascertained in further studies of comparative gene expression in tractable orthopoxviruses^34^.

The B15R_**91-101**_ peptide presented by VACV-infected HeLa cells is not recognized by mouse and human VACV-reactive CD8 + T cells^20^, but is recognized by ECTV-reactive CD8^+^ T cells. In both VACV and ECTV proteomes, B15R ORF has a six amino acid residue (IREIS**A** in VACV and IREIS**S** in ECTV) insertion after an threonine at position 97 (position 7 in B15R_91-101_). This insertion splits the epitope into two, an amino-terminal of about two-third and a carboxyl-terminal one-third, fragments (see Table S2). We predict that an enzymatic cleavage and cis-peptidation reaction within the proteasome splices out the six amino acid residue insert that abuts the two epitope fragments to generate the B15R_91-101_ epitope. Such ‘cleave and re-ligate’ reactions are observed in other MHC-I restricted antigens including virus-derived epitopes^35–39^. Why then VACV infection of HeLa cell line generated the B15R_91-101_ peptide yet why it is not recognized by VACV-reactive T cells elicited in B7.2^tg^ mice warrants further investigation.

As demonstrated in this study, some epitopes vary in their amino acid sequences, suggesting another mechanism for differential recognition of epitopes by CD8^+^ T cells elicited by different members of the orthopoxviruses. An example studied here showed that an N72K variation between the ECTV C4R protein versus the orthologous VACV B8R protein altered the presentation and recognition of the ECTV epitope. Such a variation resulted in the C4R_70–78_ peptide that bound to B7.2 poorly or not at all. Hence, it was neither immunogenic nor antigenic. It should be noted that it is unclear whether C4R is appropriately processed to generate C4R_70–78_ peptide and, hence, would need additional study. Other such examples may include N2L_104–113_ and O1L_335–344_ epitopes, which were recognised by VACV-specific but not ECTV-reactive CD8^+^ T cells. Alternatively, it is possible that the lack of recognition could be reflected in a low precursor frequency of CD8^+^ T cells against the ECTV variant of the N2L_104–113_ (Q107K) epitope. O1L_335–344_, on the other hand, is significantly different in the ECTV *O1L orf* [(Table 1); based on Netblast (blastcl3: www.ncbi.nlm.nih.gov) using ECTV txid12643] and, hence, may not be proteolytically generated, but if it is, it may not bind to B7.2 from lacking the critical proline at position 2 of the peptide that is a dominant anchor of almost all B7.2-binding peptides^40^.

To illustrate how these findings affect the design of a subunit vaccine, we engineered OVA so as to contain VACV/ECTV CD8^+^ T cell-reactive peptides (rOVA-3). This engineering disrupted the H-2A^b^-restricted epitope OT-II. The disruption of OT-II did not prevent the generation of lasting CD8^+^ T cell when mice were prime boost vaccinated with rOVA-3 plus αGalCer as the adjuvant. This result confirmed the finding from a previous report demonstrating that NKT cells—the target of αGalCer adjuvant—was sufficient, without the need for CD4^+^ T cell help to generate a robust CD8^+^ T cell response^30^. This study did not directly address whether CD4^+^ T cell help was essential for installing CD8^+^ TRM T cells within tissues such as the lungs^30^. Nonetheless, we found that i.n. subunit vaccination with rOVA-3, which lacks the CD4^+^ T cell epitope OT-II, installed CD8^+^ TRM T cells within the lungs, suggesting that this feature of the response was independent of CD4^+^ T cell help.

Most subunit vaccine design strategies utilize a mixture of individual protein antigens to deliver multiple epitopes to test efficacy in preclinical models. Here we have demonstrated the ability to elicit CD8^+^ T cell response to multiple epitopes in mice immunized with a single protein that contains three epitopes. Whilst the primed and boosted mice responded to two of the three (D1R_808–817_, B8R_70–78_, and C4R_70–78_) epitopes, by engineering a third that is known to elicit a response in place C4R_70–78_ can deliver a third epitope from rOVA. This novel strategy can be developed further to carry multiple T cell epitope cargoes within the OVA scaffold. The solubility and stability of OVA coupled with the availability of a three-dimensional structure of OVA solved by x-ray crystallography, and the knowledge of constitutive and immunoproteasome cleavage motifs can facilitate engineering multiple T cell epitopes into a single protein^41–46^. Alternatively, de novo protein engineering into nanoparticles that cage adjuvant/s can be developed into a T cell-targeted vaccine^47–49^.

Moreover, the CD8^+^ T cell responses generated in the lungs by the above vaccination strategy waned by 45 days post boost to about a fourth of the original frequency. Consistent with this decline, lethal ECTV challenge of rOVA-3 prime boost vaccinated B7.2^tg^ mice resulted in poor survival of mice (data not shown). Whether the rapid decline in antigen-specific CD8^+^ T cell response in the lungs and poor recall response upon virus challenge resulted from a lack of CD4^+^ T cell help warrants further investigations. These results collectively demonstrate the potential that the understanding of heterotypic immunity can be leveraged to generate safe and effective subunit vaccines.

## Materials and methods

### Mice and infection and immunizations

B6-*K*^*0*^*D*^*0*^;B*07:02^*tg*^ (B7.2^tg^) transgenic mice were previously described^50^. B7.2^tg^ mice were bred and maintained in H-2K^b^ and H-2D^b^ deficient background as described previously^20,50^. All mouse crosses and experiments complied with the M160174-00, V/17/002, and V1900038-00 protocols approved in accordance with relevant guidelines and regulations of Vanderbilt University Institutional Animal Care and Use Committee.

B7.2^tg^ mice were inoculated i.n. with sublethal dose of VACV Western Reserve (WR) strain (1 × 10^5^ pfu) or ECTV (10 pfu) and challenged i.n. 4 weeks later with a lethal dose of VACV (1 × 10^6^ pfu) or ECTV (200 pfu), respectively. Mock infection with PBS served as the negative control. Lungs and spleen from mock and poxvirus challenged mice were harvested after 7–10 days post challenge.

B7.2^tg^ mice were primed and boosted i.n. with 70–100 μg rOVA-3 (described below) expressing C4R_70–78_, B8R_70–78_ and D1R_808–817_ epitopes and 1 μg αGalCer (Diagnocine LLC). Lungs and spleen from mock and poxvirus challenged mice were harvested after 7–10 days post challenge.

TriVax immunizations were performed as described previously^20,51^. Briefly, B7.2^tg^ mice were injected i.v. with a mixture of 200 μg peptides (C4R_70–78_, B8R_70–78_ and D1R_808–817_, K6L_17-25_ and pHY/H-2Db; 40 μg of each peptide) that were mixed with 50 μg anti-mouse CD40 (FGK 45.5 BioxCell) and 50 μg poly-I:C (HMW, Invitrogen). A week later, mice were boosted i.p. with 2 × 10^6^ BMDCs prepared as described previously^52^ that were LPS (Sigma) maturated, and peptide pulsed (10 μg/ml of each peptide). Lungs and spleen from mock and poxvirus challenged mice were harvested after 8–10 days post challenge.

Airway cells were obtained by lung perfusion as described previously^27^. Single cell suspensions from spleen and lungs were prepared as described previously^53,54^. In some experiments, to identify CD8^+^ IST and MV TRM T cells in the lungs, mice were injected i.v. with 2 μg fluorescently labelled anti-CD45 mAb and organs harvested as described before^27,54^.

### Constructs

Constructs encoding biotin ligase (BirA), human β2-m light chain and B*07:02 (B7.2) heavy chain for expression in *E. coli*, and their production and purification have been described^20,24,27^.

A recombinant OVA (rOVA-3) protein was designed in which the original cryptic, OT-I and OT-II epitopes were replaced with C4R_70–78_, B8R_70–78_ and D1R_808–817_ epitopes, respectively (Fig. 6A). rOVA-3 containing a C-terminal hexa-histidine tag was constructed by gene synthesis and cloned into pET24a for expression in *E. coli*. Similarly, rOVA-HY was constructed by gene synthesis; the OT-II epitope in OVA is substituted by the HY peptide (Fig. S3A). Both rOVA-3 and rOVA-HY were produced and purified as above using metal affinity chromatography. The resulting protein was refolded by dialysis using the method described previously^20,27,53^. rOVA-3 was prepared fresh for each immunisation.

*Viruses*: VACV WR strain (ATCC, VR-119) was grown in and titrated with BSC-40 cells as described previously^20^. ECTV strain MOS3, a plaque purified strain grown in BS-C-1 cells and purified through a sucrose cushion^55^ and titration were performed as described previously^56^.

### Generation of peptide-HLA class I tetramers

Procedures for the production of recombinant human β2-m and B7.2, MHC-I refolding with the UV sensitive conditional peptide AARG-J-TLAM (J, 3-amino-3-(2-nitro)phenyl-propionic acid, which is a UV-labile β-amino acid residue), biotinylation and purification of refolded HLA-class I monomers, UV-mediated peptide exchange of the conditional peptide with VACV-derived peptides were performed as described previously^20,24^. VACV peptide exchanged B7.2 (pB7.2) monomers were tetramerized with either PE-, APC-, PE-Cy7-, APC-Cy7-strepatavidin (Invitrogen) or BV421-streptavidin (Biolegend) conjugated fluorochromes as described^20,24^. For dual color encoding, each pB7.2 monomers were tetramerized with two fluorochromes. Background staining was determined by irrelevant HMPV-derived CD8^+^ T cell-reactive epitope/B7.2 tetramer binding or by staining of mock infected samples.

### Measuring pHLA-I stability by ELISA

Soluble AARG-J-TLAM/B7.2 monomer was exchanged with varying concentrations of peptides. B7.2 stabilization thereupon was measured using ELISA as describes^24^. Briefly, Nunc Immuno plate 96 wells (Fisher Scientific) were coated with 2 μg/ml streptavidin overnight at 4 °C. Plates were washed with 0.05% Tween 20 in PBS (wash buffer) and blocked with 2% BSA in PBS (blocking buffer) for 1 h at room temperature (RT). pB7.2 monomers exchanged with various peptides were diluted to a final concentration of 5 nM, added (100 μl/well) in triplicates to wells bound with the pan-HLA-I mAb W6/32 after removing blocking buffer. After 1 h at RT, plates were washed with wash buffer, and 1 μg/ml anti-β2-m-HRP mAb (Novus Biologicals) was added, and incubated for 1 h at RT. Plates were washed with wash buffer, and 100 μL of 1-Step™ Ultra TMB-ELISA solution was added to each well (Fisher Scientific). Color development was visually monitored, and the reaction stopped by adding 50 μL of 2 N H_2_SO_4_. Absorbance at 450 nm was read using a BioTek Synergy HTX Plate Reader and plotted against peptide concentration used in the exchange reaction.

### Tetramer and antibody staining

All mAbs used for the study are listed in Table S3. Single cell suspensions (2–3 × 10^6^) were incubated with Ghost Voilet 510 viability dye (Tonbo Bioscience) in PBS containing 50 nM Dasatinib (LC laboratories) to differentiate between live/dead cells. After washing cells with FACS buffer (2% v/v FBS and 50 nM Dasatinib in PBS) once, cells were incubated in FACS buffer containing 0.2 μg anti-CD16/CD32 mAb for 15 min on ice to block mAbs from binding to Fc receptors. Cells were then incubated in the dark with fluorochrome-conjugated mAbs to detect surface markers. After 45 min, cells were washed twice with FACS buffer. Surface-stained cells were incubated with 0.25–0.5 μg/ml pB7.2 tetramers in PBS containing 50 nM Dasatinib. For the screening study, we adopted a dual colour encoding strategy as described^23^. With five fluorochromes, we detected 10 different specificities in a single reaction. Flow cytometric data were acquired using FACSCanto II (BD Biosciences) and FACS (.fcs) files were analysed with FlowJo software (Tree Star).

Phenotypic analysis of CD8^+^ T cells was performed by co-staining with p/B7.2 tetramers and anti-CD8α-Pacific Blue, -B220-FITC, -CD44-APC (clone IM7), -CD62L-APC-Cy7 (clone MEL-14), -CD127-PE (clone SB/14; all from BD Bioscience) or anti-KLRG-1-APC (clone 2F1; eBioscience).

### ELISpot

Splenocytes (2 × 10^6^/ml) from Trivax primed and epitope-pulsed BMDC boosted, mice^20^ were plated in triplicates and co-cultured with the indicated peptide concentrations or medium alone for 48 h. IFN-γ secreted by antigen stimulated cells were measured and quantified by ELISpot assay as previously described^28,57^ using 1–1.5 μg/ml anti-mouse IFN-γ mAb (AN18, BD Biosceinces).

### Statistics

Data comparisons were performed using Prism version 5.0 (GraphPad software). Where indicated, multiple group comparisons were performed using 1-way ANOVA with Turkey post test.

## Supplementary information

Supplementary information
