# Supplementary material for: Heterotypic immunity against vaccinia virus in an HLA-B*07:02 transgenic mousepox infection model
Source: Sci Rep. 2020 Aug 5;10:13167. doi: 10.1038/s41598-020-69897-w (PMC7406653; doi:10.1038/s41598-020-69897-w)
Supplement: Supplementary file 1 — Supplementary information [file 41598_2020_69897_MOESM1_ESM.pdf]

SUPPLEMENTRY MATERIALS

Heterotypic immunity against vaccinia virus in an HLA-B*07:02 transgenic mousepox infection model

Running title: Ectromelia virus-derived CD8+ T cell epitopes

Amrendra Kumar[1,2], Naveen Chandra Suryadevara[1,2], Kyle J. Wolf[4], John T. Wilson[3], Richard J. DiPaolo[4], James D. Brien[4], & Sebastian Joyce[1,2,5]

[1]Department of Veterans Affairs, Tennessee Valley Healthcare System; [2]Department of Pathology, Microbiology & Immunology, Vanderbilt University Medical Centre; [3]Department of Chemical & Biomolecular Engineering, Vanderbilt University; Nashville, Tennessee; [4]Department of Molecular Microbiology & Immunology, Saint Louis University School of Medicine, St. Louis, Missouri.

[5]Corresponding author: Sebastian Joyce, Ph.D., sebastian.joyce@vumc.org

TABLE S1: Properties of peptides chosen to generate binary encoded pB7.2 tetramers

	ORF/self[a]	VACV[b]	ECTV[c]	Source[d]	pB7.2 monomer exchange, %[e]
1	A24R$_{1002-1010}$	KPYASKVFF	•	MS	70.83053
2	A34R$_{82-90}$	LPRPDTRHL	•	MS	81.74804
3	A3L$_{192-200}$	SPSNHHILL	•	MS	74.94446
4	A47L$_{227-236}$	KPVSDLYTSM	•	MS	56.64893
5	A4L$_{126-135}$	APASSLLPAL	NSH	MS	76.23163
6	B17L$_{181-190}$	APYPGNVLVY	•	MS	82.74924
7	B18R$_{305-313}$	RPADSITYL	**H**P**S**DSITYL	MS	94.84093
8	B18R$_{305-313}$[f]	RPLDSITYL	**H**P**S**DSITYL	MS	83.9946
9	B8R$_{70-78}$	FPKNDFVSF	FP**N**NDFVSF	MS	94.85919
10	C10L$_{41-49}$	LPMEDNSDI	•	MS	46.47672
11	D11L$_{506-514}$	MPTVDEDLF	•	MS	58.24184
12	D5R$_{375-383}$	LPKEYSSEL	•	MS	74.42226
13	D9R$_{26-35}$	IPRSKDTHVF	•	MS	84.6781
14	E1L$_{10-18}$	LPNITLKII	•	MS	25.29541
15	E2L$_{216-224}$	RPRDAIRFL	•	MS	71.43947
16	E9L$_{175-183}$	FPSVFINPI	•	MS	57.55645
17	E9L$_{175-183}$[f]	FPSVFINPV	FPSVFINP**I**	MS	73.84057
18	E9L$_{526-534}$	FPYEGGKVF	•	MS	111.9869

19	G5R341-349	LPCQLMYAL	•	MS	70.8617
20	G7L175-183	LPMIIGEPI	•	MS	70.068
21	H1L65-73	LPNSNINII	•	MS	59.41963
22	H5R89-97	SPSPGVGDI	NSH	MS	54.39731
23	I1L53-62	IPVDLVKSSF	•	MS	46.01204
24	I4L498-507	RPIGIGVQGL	•	MS	70.52276
25	I6L237-245	FPTNTLTSI	•	MS	57.23258
26	I6L272-280	IPKKIVSLL	•	MS	56.66139
27	I7L342-350	TPPKSFKSL	•	MS	57.33455
28	J3R8-16	KPFMYFEEI	•	MS	42.75962
29	K1L151-159	IPSTFDLAI	•	MS	48.3324
30	K6L17-25	KPITYPKAL	•	MS	78.04875
31	L4R37-45	FPRSMLSIF	•	MS	40.13252
32	N2L104-113	RPNQHHTIDL	RPN**K**HHTIDL	MS	86.15705
33	I4L670-678	LPEDIKRVY	•	MS	46.96676
34	D13L160-168	TPFDVEDTF	•	MS	52.06957
35	TA25R954-962	FPSSNETSI	•	MS	49.48632
36	D1R808-817	RPSTRNFFEL	•	MS	70.72724
37	B15R91-101	IPDEQKTIIGL	•	Predicted	76.69625
38	O1L549-557	IPITDSLSF	•	Public	64.66695
39	O1L549-557[f]	IPITESLSF	IPIT**D**SLSF	Public	58.66162

40	B22R$_{72-80}$	TVADVRHCL	**SID**DVR**Q**CL	Public	79.57025
41	C1L$_{102-111}$	KPKPAVRFAI	**EYNR**AVRFA**E**	Public	75.30461
42	D1R$_{686-694}$	HPRHYATVM	•	Public	71.18591
43	F4L$_{6-14}$	APNPNRFVI	•	Public	98.25802
44	J6R$_{303-311}$	MPAYIRNTL	•	Public	86.58602
45	O1L$_{335-344}$	RPMSLRSTII	RP**I**SLRSTII	Public	74.84205
46	J2R$_{116-124}$	KPFNNILNL	•	Public	56.809954
47	TTF2$_{333-341}$	APAAPGLSL	na	Self	76.23394
48	HNRPM$_{499-507}$	APIDRVGQTL	na	Self	72.42752
49	SAP145$_{18-27}$	APIQGNREEL	na	Self	69.33693
50	HK2$_{904-912}$	APKCDVSFL	na	Self	59.89381
51	CUL-4A$_{5-14}$	APRKGSFSAL	na	Self	95.3412
52	ABCC12$_{1347-1353}$	APSAFGMLL	na	Self	67.52423
53	NLRP2$_{213-220}$	GPSGLGKTAI	na	Self	60.51939
54	TRIM27$_{468-476}$	GPVRPYFSL	na	Self	78.9362
55	FASN$_{1169-1178}$	IPRDPSQQEL	na	Self	77.31273
56	CDCA7$_{94-103}$	KPRPDVTNEL	na	Self	77.95413
57	hCG1980884$_{1807-1815}$	KPYFPPRIL	na	Self	62.56214
58	FASN$_{925-933}$	LPKTGTVSL	na	Self	74.5599
59	RACGAP1$_{465-473}$	LPQANRDTL	na	Self	100.5323
60	COPS8$_{163-172}$	LPRKPVAGAL	na	Self	99.14703

61	PACSIN2$_{337-345}$	LPSKPSSTL	na	Self	66.27471
62	FUS/BBF2H7$_{91-100}$	LPVSCTPGPL	na	Self	56.58131
63	TYMS$_{274-282}$	RPFPKLRIL	na	Self	64.86887
64	VEGFR3$_{1050-1058}$	RPGSSDRVL	na	Self	86.67953
65	UROD$_{163-171}$	RPQASHQLL	na	Self	65.97249
66	HECTD1439-447	RPQVAKTLL	na	Self	105.5192
67	SIK1$_{430-447}$	RPRPVSPSSL	na	Self	78.38857
68	TBX1$_{442-450}$	RPSPPNPEL	na	Self	74.64187
69	LOC399706$_{139-145}$	SPAGSTRVL	na	Self	82.34095
70	SLC37A3$_{377-386}$	SPNDKSINAL	na	Self	79.91597
71	CA9$_{397-406}$	SPRAAEPVQL	na	Self	83.77609
72	KCDT10$_{28-36}$	SPSSKYVKL	na	Self	89.20886
73	TNKS$_{142-150}$	SPSSPGSSL	na	Self	78.06154
74	MAP1S$_{945-954}$	SPVYLDLAYL	na	Self	42.82756
75	HRT3$_{162-170}$	TPSNTPTGPL	na	Self	52.25194

[a]Open reading frames (ORFs)/source molecules and location of epitopes defined based on Copenhagen reference strain (VACCC, txid 10249), homo sapiens (NCBITaxon:9606)

[b]amino acid sequence based on Copenhagen reference strain (VACCC, ID 10249)

[c]Amino acid changes in the orthologous ECTV peptide; ●, conserved sequences with 100% homology; NSH, no significant homology; red bold residues, variation in ECTV within the VACV-derived epitope; based on Netblast (blastcl3: www.ncbi.nlm.nih.gov) using ECTV txid12643; na, not applicable

[d]MS, mass spectrometry discovered VACV peptides discovered by us[1], predicted, reported as predicted VACV epitope; public, reported as positive in human or HLA class I transgenic mouse $CD8^+$ T cell assay; self, naturally processed self peptides[2]

[e]Average rescue of pB7.2 monomer assessed in an UV-mediated peptide exchange assay[1]; see Materials and Methods and Figure 5C; a threshold of $\geq$40% exchange was used to select peptides for tetramer binding study

[f]VARV orthologue, txid 10255 variola virus

Table S2: Homology of VACV epitopes uniquely recognised by ECTV-reactive CD8$^+$ T cells

Orf: I6L[a,b]; epitope: I6L$_{272-281}$

```
ECTV   1    MNNFVKQVASKSLKPTKKLSPSDEVISLNECIISFNLDNFYYCNDGLFTKPINTPEDVLK  60
            MNNFVKQVASKSLKPTKKLSPSDE ISLNECIISFNLDNFYYCNDGLFTKPINTPEDVLK
VACV   1    MNNFVKQVASKSLKPTKKLSPSDEAISLNECIISFNLDNFYYCNDGLFTKPINTPEDVLK  60

ECTV   61   SLLIMESFAYEKMIIKGLIKILISRAYINDIYFTPFGWLTGVDDDPETHVVIKIIFNSSL  120
            SLL+MESFAYEKMIIKGLIKILISRAYINDIYFTPFGWLTGVD DPETHVVIKIIFNSSL
VACV   61   SLLVMESFAYEKMIIKGLIKILISRAYINDIYFTPFGWLTGVDYDPETHVVIKIIFNSSL  120

ECTV   121  ISIKSQVIEYLKPYNVNNLSVLTTEKELSINTFNVPDSIPMSIISFFPFDTDFILVILFF  180
            ISIKSQVIEYLKPYNVNNLSVLTTEKELSINTFNVPDSIPMSIISFFPFDTDFILVILFF
VACV   121  ISIKSQVIEYLKPYNVNNLSVLTTEKELSINTFNVPDSIPMSIISFFPFDTDFILVILFF  180

ECTV   181  GVYNDSYCGISYISPKERLPYIIEILKPLVSEINMLSDEIGRTSSIRIFNSTSVKKFPTN  240
            GVYNDSYCGISYISPKERLPYIIEILKPLVSEINMLSDEIGRTSSIRIFNSTSVKKFPTN
VACV   181  GVYNDSYCGISYISPKERLPYIIEILKPLVSEINMLSDEIGRTSSIRIFNSTSVKKFPTN  240

ECTV   241  TLTSICEIVYSFDESSFPTPKTFTPLNASPYIPKKIVSLLDLPSNVEIKAISRGGVDFIT  300
            TLTSICEIVYSFDES FPTPKTFTPLNASPYIPKKIVSLLDLPSNVEIKAISRGGVDFIT
VACV   241  TLTSICEIVYSFDESFFPTPKTFTPLNASPYIPKKIVSLLDLPSNVEIKAISRGGVDFIT  300

ECTV   301  HINNKRLNTILVIAKDNFLKNSTFSGTFIKENIIWKGIYTYRIIKSSFPVPTIKSVTNKK  360
            HINNKRL TILVIAKDNFLKNSTFSGTFIKENIIWKGIYTYRIIKSSFPVPTIKSVTNKK
VACV   301  HINNKRLTTILVIAKDNFLKNSTFSGTFIKENIIWKGIYTYRIIKSSFPVPTIKSVTNKK  360

ECTV   361  KICKKHCFVNSQYTTRTLSHIL  382
            KICKKHCFVNSQYTTRTLSHIL
VACV   361  KICKKHCFVNSQYTTRTLSHIL  382
```

Orf: B15R[a,b]; epitope: B15R$_{91-101}$ (C8R in ECTV

```
ECTV   1    MTANFSTHVFSPQHCGCDRLTSIDDVRQCLTEYIYWSSYAYRNRQCAGQLYSTLLSFRDD 75
            MTANFSTHVFSPQHCGCDRLTSIDDV+QCLTEYIYWSSYAYRNRQCAGQLYSTLLSFRDD
VACV   1    MTANFSTHVFSPQHCGCDRLTSIDDVKQCLTEYIYWSSYAYRNRQCAGQLYSTLLSFRDD 60

ECTV   76   AESVFIDIRELVKNMPWDDVKDCAEIIRCYIPDEQKTIREISSIIGLCAYAATYWGGEDH  135
            AE VFIDIRELVKNMPWDDVKDC EIIRCYIPDEQKTIREIS+IIGLCAYAATYWGGEDH
VACV   61   AELVFIDIRELVKNMPWDDVKDCTEIIRCYIPDEQKTIREISAIIGLCAYAATYWGGEDH  120

ECTV   136  PTSNSLNALFVMLELLNYVDYNIIFRRMN  164
            PTSNSLNALFVMLE+LNYVDYNIIFRRMN
VACV   121  PTSNSLNALFVMLEMLNYVDYNIIFRRMN  149
```

Note IREISS insertion at position 91 in ECTV[b] but lacking in this strain of VACV[c]

```
ECTV    1   MTANFSTHVFSPQHCGCDRLTSIDDVRQCLTEYIYWSSYAYRNRQCAGQLYSTLLSFRDD  75
            MTANFSTHVFSPQHCGCDRLTSIDDV+QCLTEYIYWSSYAYRNRQCAGQLYSTLLSFRDD
VACV    1   MTANFSTHVFSPQHCGCDRLTSIDDVKQCLTEYIYWSSYAYRNRQCAGQLYSTLLSFRDD  60
```

```
ECTV   76    AESVFIDIRELVKNMPWDDVKDCAEIIRCYIPDEQKTIREISSIIGLCAYAATYWGGEDH  135
             AE VFIDIRELVKNMPWDDVKDC EIIRCYIPDEQKT      IIGLCAYAATYWGGEDH
VACV   61    AELVFIDIRELVKNMPWDDVKDCVEIIRCYIPDEQKT------IIGLCAYAATYWGGEDH  114

ECTV   136   PTSNSLNALFVMLELLNYVDYNIIFRRMN  164
             PTSNSLNALFVMLE+LNYVDYNIIFRRMN
VACV   115   PTSNSLNALFVMLEMLNYVDYNIIFRRMN  143
```

Orf: D9R[a,b]; epitope: D9R$_{26-35}$

```
ECTV   1     MGITMDEEVIFETPRELISIKRIKDIPRSKDTHVFAACITSDGYPLIGARRTSFAFQAIL  60
             MGITMDEEVIFETPRELISIKRIKDIPRSKDTHVFAACITSDGYPLIGARRTSFAFQAIL
VACV   1     MGITMDEEVIFETPRELISIKRIKDIPRSKDTHVFAACITSDGYPLIGARRTSFAFQAIL  60

ECTV   61    SQQNSDSIFRVSTKLLRFMYYNELREIFRRLRKGSINNIDPHFEELILLGGKLDKKESIK  120
             SQQNSDSIFRVSTKLLRFMYYNELREIFRRLRKGSINNIDPHFEELILLGGKLDKKESIK
VACV   61    SQQNSDSIFRVSTKLLRFMYYNELREIFRRLRKGSINNIDPHFEELILLGGKLDKKESIK  120
                89                                     125
ECTV   121   DCLKRELKEESDERITVKEFGNVILKLTTRDKLFNKVYIGYCMACFINQSLEDLSHTSIY  180
             DCL+RELKEESDERITVKEFGNVILKLTTRDKLFNKVYI YCMACFINQSLEDLSHTSIY
VACV   121   DCLRRELKEESDERITVKEFGNVILKLTTRDKLFNKVYISYCMACFINQSLEDLSHTSIY  180

ECTV   181   NVEIRKIKSLNDCINDDKYEYLSYIYNMLVNSK  213
             NVEIRKIKSLNDCINDDKYEYLSYIYNMLVNSK
VACV   181   NVEIRKIKSLNDCINDDKYEYLSYIYNMLVNSK  213
```

Orf: A24R[,b]; epitope: A24R$_{1002-1010}$

```
ECTV   1     MKKNTDSEMDQRLGYKFLVPDPKAGVFYRPLHFQYVSYSNFILHRLHEILTVKRPLLSFK  60
             MKKNTDSEMDQRLGYKFLVPDPKAGVFYRPLHFQYVSYSNFILHRLHEILTVKRPLLSFK
VACV   1     MKKNTDSEMDQRLGYKFLVPDPKAGVFYRPLHFQYVSYSNFILHRLHEILTVKRPLLSFK  60

ECTV   61    NNTERIMIEISNVKVTPPDYSPIIASIKGKSYDALATFTVNIFKEVMTKEGISITKISSY  120
             NNTERIMIEISNVKVTPPDYSPIIASIKGKSYDALATFTVNIFKEVMTKEGISITKISSY
VACV   61    NNTERIMIEISNVKVTPPDYSPIIASIKGKSYDALATFTVNIFKEVMTKEGISITKISSY  120

ECTV   121   EGKDSHLIKIPLLIGYGNKNPLDTAKYLVPNVIGGVFINKQSVEKVGINLVEKITTWPKF  180
             EGKDSHLIKIPLLIGYGNKNPLDTAKYLVPNVIGGVFINKQSVEKVGINLVEKITTWPKF
VACV   121   EGKDSHLIKIPLLIGYGNKNPLDTAKYLVPNVIGGVFINKQSVEKVGINLVEKITTWPKF  180

ECTV   181   RVVKPNSFTFSFSSVSPPNVLPTRYRHYKISLDISQLEASNISSTKTFITVNIVLLSQYL  240
             RVVKPNSFTFSFSSVSPPNVLPTRYRHYKISLDISQLEA NISSTKTFITVNIVLLSQYL
VACV   181   RVVKPNSFTFSFSSVSPPNVLPTRYRHYKISLDISQLEALNISSTKTFITVNIVLLSQYL  240

ECTV   241   SRVSLEFIRRSLSYDMPPEVVYLVNAIIDSAKRITESITDFNIDTYINDLVEAEHIKQKS  300
             SRVSLEFIRRSLSYDMPPEVVYLVNAIIDSAKRITESITDFNIDTYINDLVEAEHIKQKS
VACV   241   SRVSLEFIRRSLSYDMPPEVVYLVNAIIDSAKRITESITDFNIDTYINDLVEAEHIKQKS  300

ECTV   301   QLTINEFKYEMLHNFLPHMNYTPDQLKGFYMISLLRKFLYCIYHTSRYPDRDSMVCHRIL  360
             QLTINEFKYEMLHNFLPHMNYTPDQLKGFYMISLLRKFLYCIYHTSRYPDRDSMVCHRIL
VACV   301   QLTINEFKYEMLHNFLPHMNYTPDQLKGFYMISLLRKFLYCIYHTSRYPDRDSMVCHRIL  360
```

```
ECTV    361   TYGKYFETLAHDELENYIGNIRNDIMNNHKNRGTYAVNIHVLTTPGLNHAFSSLLSGKFK   420
              TYGKYFETLAHDELENYIGNIRNDIMNNHKNRGTYAVNIHVLTTPGLNHAFSSLLSGKFK
VACV    361   TYGKYFETLAHDELENYIGNIRNDIMNNHKNRGTYAVNIHVLTTPGLNHAFSSLLSGKFK   420

ECTV    421   KSDGSYRTHPHYSWMQNISIPRSVGFYPDQVKISKMFSVRKYHPSQYLYFCSSDVPERGP   480
              KSDGSYRTHPHYSWMQNISIPRSVGFYPDQVKISKMFSVRKYHPSQYLYFCSSDVPERGP
VACV    421   KSDGSYRTHPHYSWMQNISIPRSVGFYPDQVKISKMFSVRKYHPSQYLYFCSSDVPERGP   480

ECTV    481   QVGLVSQLSVLSSITNILTSEYLDLEKKICEYIRSYYKDDISYFETGFPITIENALVASL   540
              QVGLVSQLSVLSSITNILTSEYLDLEKKICEYIRSYYKDDISYFETGFPITIENALVASL
VACV    481   QVGLVSQLSVLSSITNILTSEYLDLEKKICEYIRSYYKDDISYFETGFPITIENALVASL   540

ECTV    541   NPNMICDFVTDFRRRKRMGFFGNLEVGITLVRDHMNEIRINIGAGRLVRPFLVVDNGELM   600
              NPNMICDFVTDFRRRKRMGFFGNLEVGITLVRDHMNEIRINIGAGRLVRPFLVVDNGELM
VACV    541   NPNMICDFVTDFRRRKRMGFFGNLEVGITLVRDHMNEIRINIGAGRLVRPFLVVDNGELM   600

ECTV    601   MDVCQELESRLDDMTFSDIQKEFPHVIEMVDIEQFTFSNVCESVQKFRMMSKDERKQYDL   660
              MDVC ELESRLDDMTFSDIQKEFPHVIEMVDIEQFTFSNVCESVQKFRMMSKDERKQYDL
VACV    601   MDVCPELESRLDDMTFSDIQKEFPHVIEMVDIEQFTFSNVCESVQKFRMMSKDERKQYDL   660

ECTV    661   CDFPAEFRDGYVASSLVGINHNSGPRAILGCAQAKQAISCLSSDIRNKIDNGIHLMYPER   720
              CDFPAEFRDGYVASSLVGINHNSGPRAILGCAQAKQAISCLSSDIRNKIDNGIHLMYPER
VACV    661   CDFPAEFRDGYVASSLVGINHNSGPRAILGCAQAKQAISCLSSDIRNKIDNGIHLMYPER   720

ECTV    721   PIVISKALETSKIAANCFGQHVTIALMSYKGINQEDGIIIKKQFIQRGGLDIVTAKKHQV   780
              PIVISKALETSKIAANCFGQHVTIALMSYKGINQEDGIIIKKQFIQRGGLDIVTAKKHQV
VACV    721   PIVISKALETSKIAANCFGQHVTIALMSYKGINQEDGIIIKKQFIQRGGLDIVTAKKHQV   780

ECTV    781   EIPLENFNNKERDRSNAYSKLESNGLVRLNAFLESGDAMARNISSRTLEDDFARDNQISF   840
              EIPLENFNNKERDRSNAYSKLESNGLVRLNAFLESGDAMARNISSRTLEDDFARDNQISF
VACV    781   EIPLENFNNKERDRSNAYSKLESNGLVRLNAFLESGDAMARNISSRTLEDDFARDNQISF   840

ECTV    841   DVSEKYTDMYKSRVERVQVELTDKVKVRVLTMKERRPILGDKFTTRTSQKGTVAYIADET   900
              DVSEKYTDMYKSRVERVQVELTDKVKVRVLTMKERRPILGDKFTTRTSQKGTVAY+ADET
VACV    841   DVSEKYTDMYKSRVERVQVELTDKVKVRVLTMKERRPILGDKFTTRTSQKGTVAYVADET   900

ECTV    901   ELPYDENGITPDVIINSTSIFSRKTISMLIEVILTAAYSAKPYNNNGENRPVCFPSSNET   960
              ELPYDENGITPDVIINSTSIFSRKTISMLIEVILTAAYSAKPYNN GENRPVCFPSSNET
VACV    901   ELPYDENGITPDVIINSTSIFSRKTISMLIEVILTAAYSAKPYNNKGENRPVCFPSSNET   960

ECTV    961   SIDTYMQFAKQCYEHSNPQLTDDELSDKVFCEKILYDPETDKPYASKVFFGPIYYLRLRH   1020
              SIDTYMQFAKQCYEHSNP+L+D+ELSDK+FCEKILYDPETDKPYASKVFFGPIYYLRLRH
VACV    961   SIDTYMQFAKQCYEHSNPKLSDEELSDKIFCEKILYDPETDKPYASKVFFGPIYYLRLRH   1020

ECTV    1021  LTQDKATVRCRGKKTKLIRQANEGRKRGGGIKFGEMERDCLIAHGAANTITEVLKDSEED   1080
              LTQDKATVRCRGKKTKLIRQANEGRKRGGGIKFGEMERDCLIAHGAANTITEVLKDSEED
VACV    1021  LTQDKATVRCRGKKTKLIRQANEGRKRGGGIKFGEMERDCLIAHGAANTITEVLKDSEED   1080

ECTV    1081  YQDVYICENCGDIAAQIKGINTCLRCSKLNLSPLLTKIDTTHVSKVFLTQMNARGVKVKL   1140
              YQDVY+CENCGDIAAQIKGINTCLRCSKLNLSPLLTKIDTTHVSKVFLTQMNARGVKVKL
VACV    1081  YQDVYVCENCGDIAAQIKGINTCLRCSKLNLSPLLTKIDTTHVSKVFLTQMNARGVKVKL   1140
```

ECTV	1141	DFERRPPSFYK**Q**LDKVDLKPSFL	1163
		DFERRPPSFYK LDKVDLKPSFL	
VACV	1141	DFERRPPSFYK**P**LDKVDLKPSFL	1163

[a]VACV txid: 10249

[b]ECTV txid: 12643

[c]VACV txid: 10245

Blue bold, variation outside the epitope; red bold, amino acid sequence of the epitope

TABLE S3. List of Antibodies

Antigen	Fluorochrome	Clone	Source	Catalogue number
B220	FITC	RA3-6B2	BD Biosciences	553088
B220	PerCP-Cy5.5	RA3-6B2	Biolegend	103236
B220	APC-Cy7	RA3-6B2	BD Biosciences	552094
CD69	APC	H1.2F3	Biolegend	104514
CD8a	FITC	53-6.7	BD Biosciences	553031
CD8a	PerCP-Cy5.	53-6.7	BD Biosciences	551162
CD45.2	FITC	104	Biolegend	109806
CD45.2	APC	104	Tonbo Biosciences	20-0454-U100
CXCR3	APC	CXCR3-173	BD Biosciences	562266
CD62L	PE	MEL-14	BD Biosciences	553151
KLRG1	APC	2F1/KLRG1	Biolegend	138412
CD49a	PE	Ha31/8	BD Biosciences	562115
CCR8	APC	SA214G2	Biolegend	150309
CXCR6	PE	SA051D1	Biolegend	151103
CD103	PE	2E7	Biolegend	121405
Ghost Voilet 510	V510	na	Tonbo Biosciences	13-0870-T100
pB7.2 tetramer	PE	na	in house	na
pB7.2 tetramer	APC	na	in house	na
pB7.2 tetramer	BV421	na	in house	na
pB7.2 tetramer	PE-Cy7	na	in house	na
pB7.2 tetramer	APC-Cy7	na	in house	na

na, not applicable

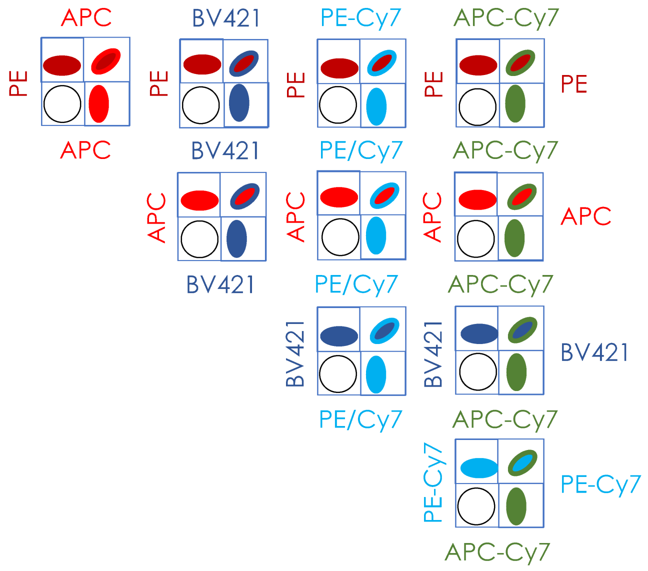

Figure S1: A schematic rendition of binary encoding of T cell epitope specificity. pMHC monomers are made to tetramers by reacting the biotinylated monomer of a single antigen specificity with two distinct fluorophore-conjugated streptavidin in two separate tubes. When a pMHC monomer of a single specificity is individually tetramerised with five different fluorophore-conjugated streptavidin molecules, and two tetramers made of two different fluorophore-conjugated streptavidin molecules mixed —and, thereby, binary encoded— one will obtain ten unique combinations as shown in the schematic. For a real example, see Figure 1A. In a variation of this binary encoding of a pMHC monomer with a single specificity, monomers of ten different specificities can be binary encoded with two unique combinations of fluorophores just with 5 different fluorophore-conjugated streptavidin molecules. For a real example, see Figure 1B. refer to refs.[3, 4] for detailed description and protocols.

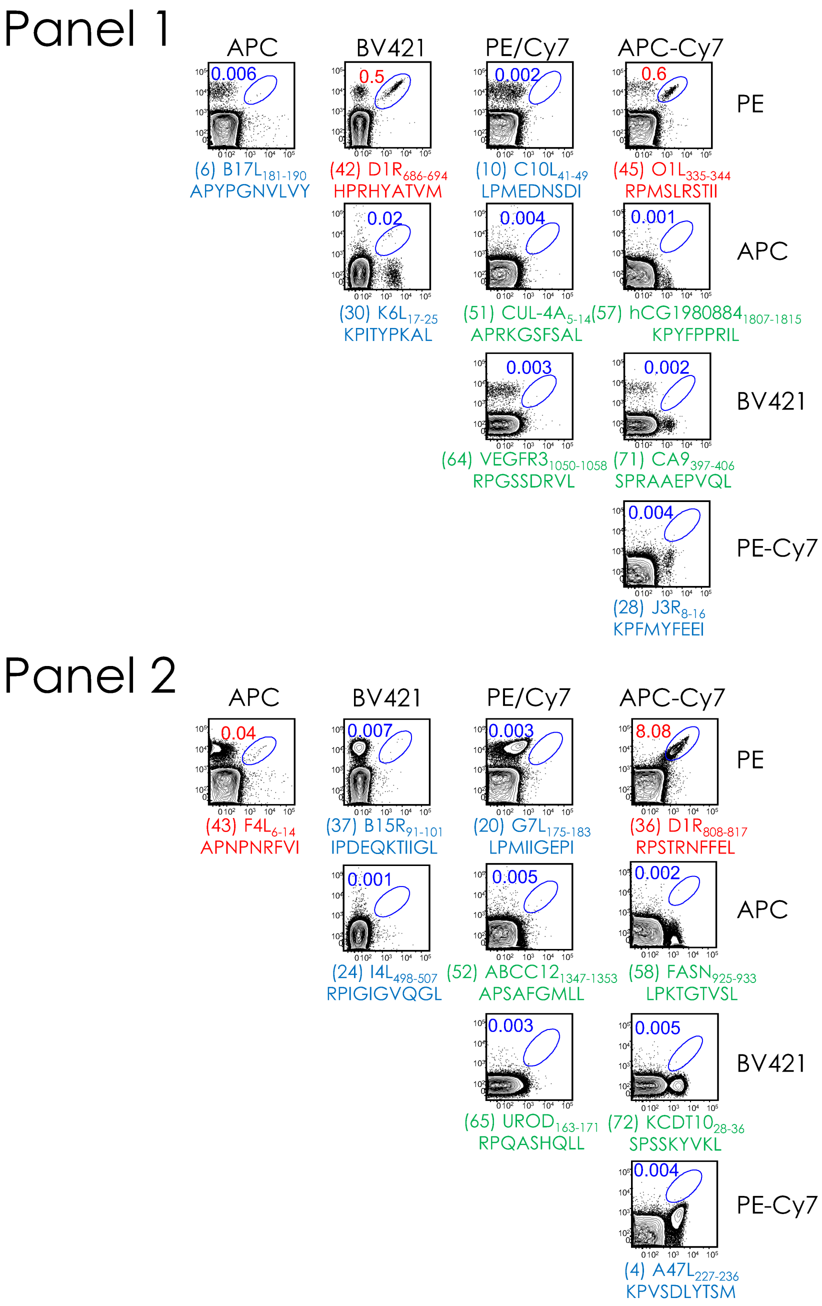

Figure S2, Panels 1&2: Tracking VACV-reactive CD8⁺ T cell responses in B7.2^{tg} mice. As in Figure 2, B7^{tg} mice were inoculated i.n. with sublethal dose of ECTV, and after 4 weeks challenged i.n. with a lethal dose of the same virus. See Materials & Methods. Spleens were harvested from mock (n=2) and ECTV (*n*=*8*) inoculated mice after 8—10 days post inoculation. CD8$^+$ T cells were identified as CD8$^+$ splenocytes after gating out dead cells and B220$^+$ cells. Peptide-specific CD8$^+$ T cells were identified using dual fluorochrome-labelled pB7.2 tetramers and binary encoding strategy as schematised in Figure S2. Red, positive VACV pB7.2 tetramer staining; blue, no staining with VACV pB7.2 tetramer; green, self p/B7.2 tetramers. *Note that Panel 3 is shown in Figure 1B.*

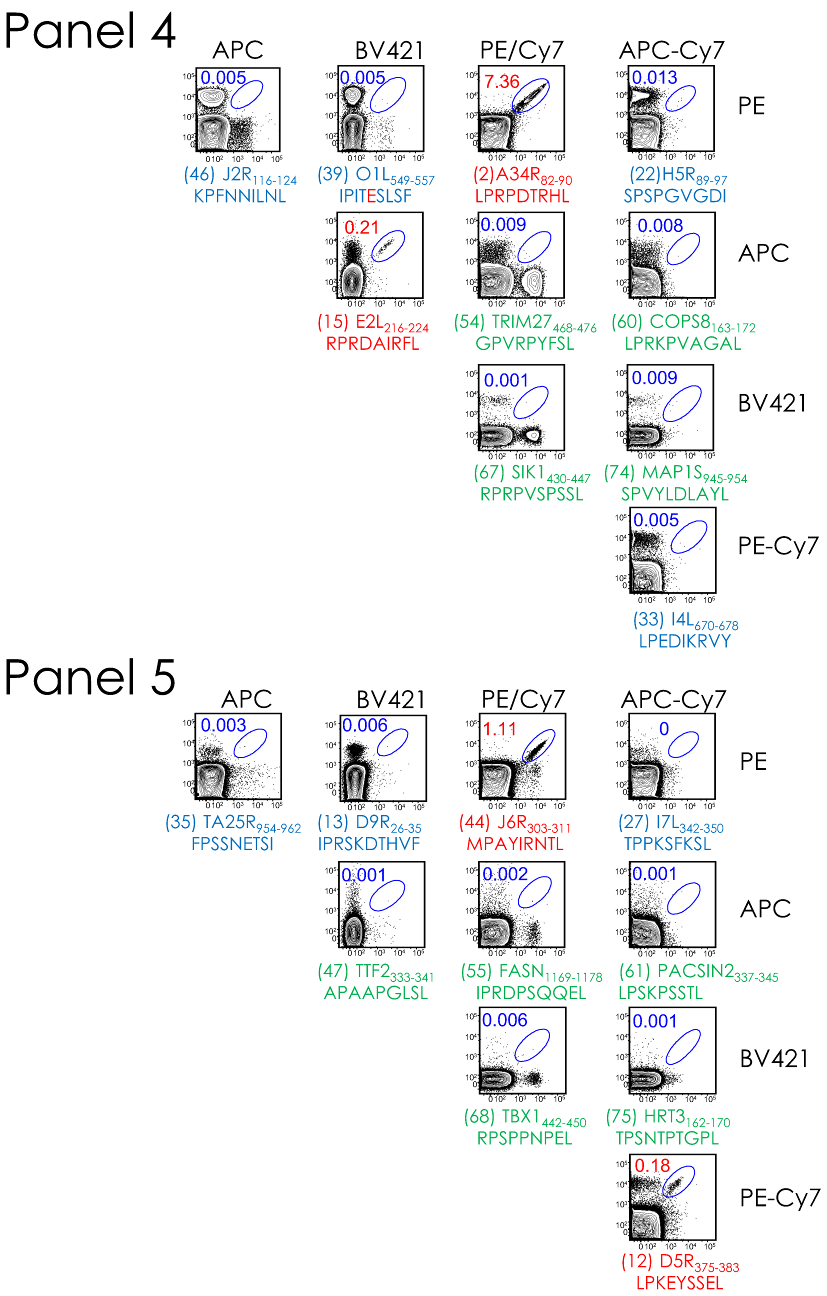

Figure S2 *continued*, Panels 4&5: Tracking ECTV-reactive CD8$^+$ T cell responses in B7.2^{tg} mice. As in Figure 2, B7^{tg} mice were inoculated i.n. with sublethal dose of ECTV, and after 4 weeks challenged i.n. with a lethal dose of the same virus. See Materials & Methods. Spleens were harvested from mock (n=2) and ECTV (*n=8*) inoculated mice after 8—10 days post inoculation. CD8$^+$ T cells were identified as CD8$^+$ splenocytes after gating out dead cells and B220$^+$ cells. Peptide-specific CD8$^+$ T cells were identified using dual fluorochrome-labelled pB7.2 tetramers and binary encoding strategy as schematised in Figure S2. Red, positive VACV pB7.2 tetramer staining; blue, no staining with VACV pB7.2 tetramer; self p/B7.2 tetramers. *Note that Panel 3 is shown in Figure 1B.*

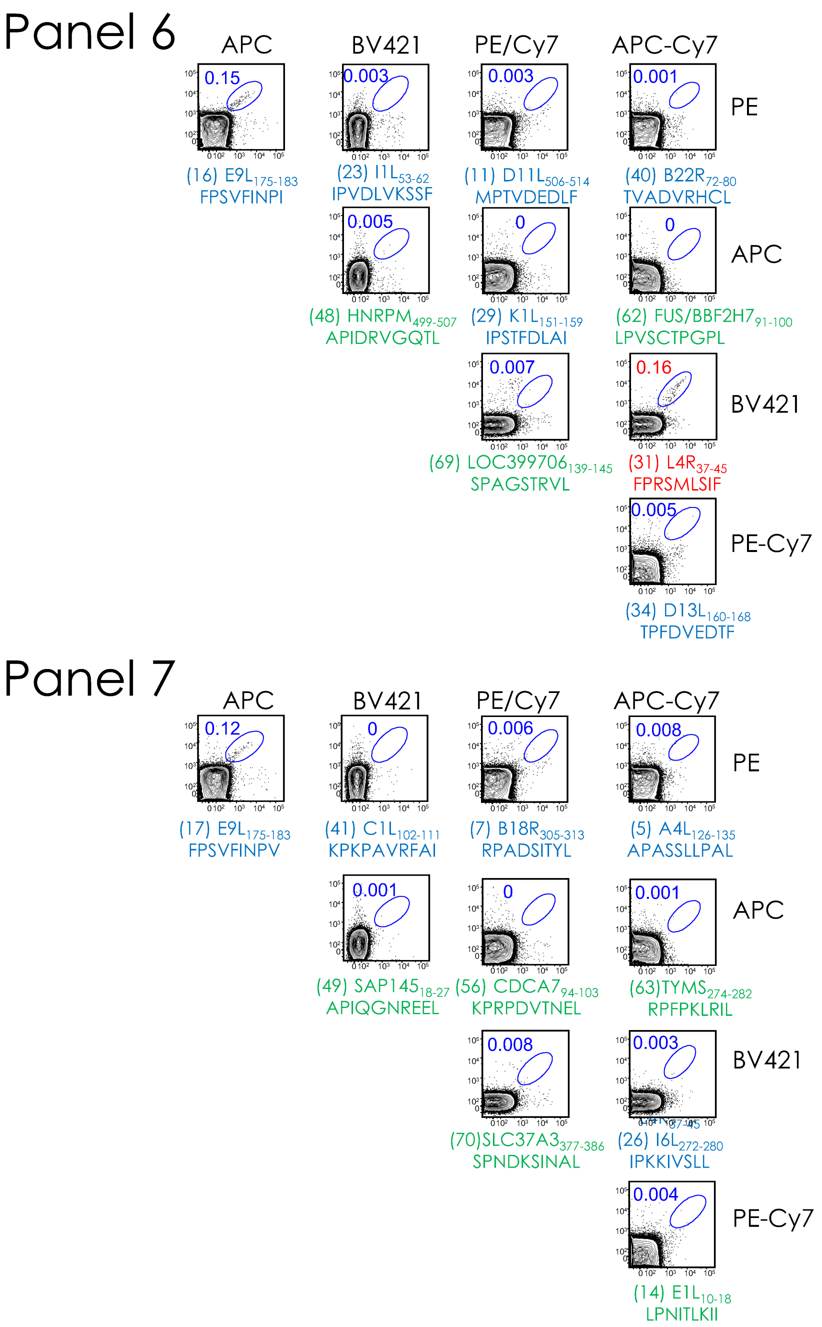

Figure S2 *continued*, Panels 6&7: Tracking ECTV-reactive $CD8^+$ T cell responses in $B7.2^{tg}$ mice. As in Figure 2, $B7^{tg}$ mice were inoculated i.n. with sublethal dose of ECTV, and after 4 weeks challenged i.n. with a lethal dose of the same virus. See Materials & Methods. Spleens were harvested from mock (n=2) and ECTV (*n*=*8*) inoculated mice after 8—10 days post inoculation. $CD8^+$ T cells were identified as $CD8^+$ splenocytes after gating out dead cells and $B220^+$ cells. Peptide-specific $CD8^+$ T cells were identified using dual fluorochrome-labelled pB7.2 tetramers and binary encoding strategy as schematised in Figure S2. Red, positive VACV pB7.2 tetramer staining; blue, no staining with VACV pB7.2 tetramer; green, self p/B7.2 tetramers.

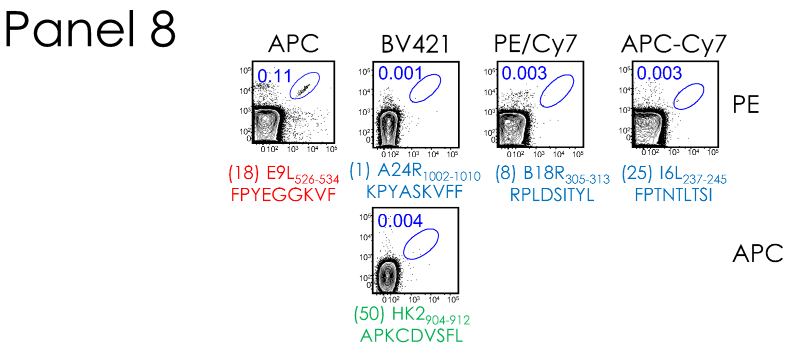

Figure S2 *continued*, Panel 8: Tracking ECTV-reactive CD8^{+} T cell responses in B7.2^{tg} mice. As in Figure 2, B7^{tg} mice were inoculated i.n. with sublethal dose of ECTV, and after 4 weeks challenged i.n. with a lethal dose of the same virus. See Materials & Methods. Spleens were harvested from mock (n=2) and ECTV (*n=8*) inoculated mice after 8—10 days post inoculation. CD8^{+} T cells were identified as CD8^{+} splenocytes after gating out dead cells and B220^{+} cells. Peptide-specific CD8^{+} T cells were identified using dual fluorochrome-labelled pB7.2 tetramers and binary encoding strategy as schematised in Figure S2. Red, positive VACV pB7.2 tetramer staining; blue, no staining with VACV pB7.2 tetramer; green, self p/B7.2 tetramers.

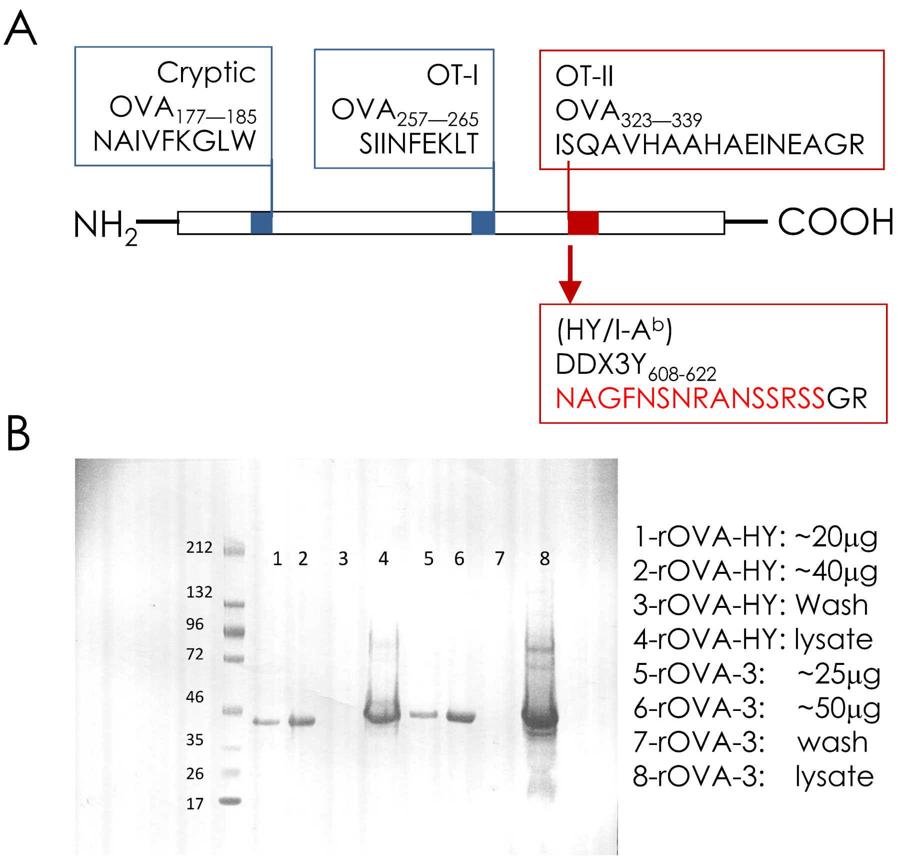

Figure S3: Design of rOVA-HY protein, and the expression and purification of rOVA-HY and rOVA-3 proteins. (**A**) Schematic rendition of the rOVA-HY construct in which the OT-II epitope was replaced with the H-2I-A^b-restricted HY epitope (NAGFNSNRAN-SSRSS) recognised by $CD4^+$ T cells elicited by C57BL/6 female mice immunised with C57BL/6 male splenocytes.[5, 6] Original cryptic, and OT-I epitopes were intact. rOVA-HY containing a C-terminal hexa-histidine tag was constructed by gene synthesis and cloned into pET24a for expression in *E. coli*. (**B**) Representative SDS/4—12%-PAGE image showing expression of indicated rOVA protein before and after purification over metal affinity column (see Materials and Methods for details).

LITERATURE CITED

1. Gilchuk, P. *et al.* Discovering naturally processed antigenic determinants that confer protective T cell immunity. *The Journal of clinical investigation* **123**, 1976-1987 (2013).

2. Spencer, C.T. *et al.* Viral infection causes a shift in the self peptide repertoire presented by human MHC class I molecules. *Proteomics Clin Appl* **9**, 1035-1052 (2015).

3. Hadrup, S.R. *et al.* Parallel detection of antigen-specific T-cell responses by multidimensional encoding of MHC multimers. *Nature methods* **6**, 520-526 (2009).

4. Andersen, R.S. *et al.* Parallel detection of antigen-specific T cell responses by combinatorial encoding of MHC multimers. *Nature protocols* **7**, 891-902 (2012).

5. Dragovic, S.M. *et al.* Proteasomes, TAP, and endoplasmic reticulum-associated aminopeptidase associated with antigen processing control CD4+ Th cell responses by regulating indirect presentation of MHC class II-restricted cytoplasmic antigens. *J Immunol* **186**, 6683-6692 (2011).

6. Scott, D. *et al.* Dendritic cells permit identification of genes encoding MHC class II-restricted epitopes of transplantation antigens. *Immunity* **12**, 711-720 (2000).
